# Multi-Scale Spatial Analysis of the Tumor Microenvironment Reveals Features of Cabozantinib and Nivolumab Efficacy in Hepatocellular Carcinoma

**DOI:** 10.3389/fimmu.2022.892250

**Published:** 2022-05-13

**Authors:** Haoyang Mi, Won Jin Ho, Mark Yarchoan, Aleksander S. Popel

**Affiliations:** ^1^ Department of Biomedical Engineering, Johns Hopkins University School of Medicine, Baltimore, MD, United States; ^2^ Sidney Kimmel Comprehensive Cancer Center, Johns Hopkins University School of Medicine, Baltimore, MD, United States; ^3^ Bloomberg~Kimmel Institute for Cancer Immunotherapy, Johns Hopkins University School of Medicine, Baltimore, MD, United States

**Keywords:** tumor-immune microenvironments (TIME), hepatocellular carcinoma, immunotherapy, biomarker, computational biology, systems biology

## Abstract

**Background:**

Concomitant inhibition of vascular endothelial growth factor (VEGF) and programmed cell death protein 1 (PD-1) or its ligand PD-L1 is a standard of care for patients with advanced hepatocellular carcinoma (HCC), but only a minority of patients respond, and responses are usually transient. Understanding the effects of therapies on the tumor microenvironment (TME) can provide insights into mechanisms of therapeutic resistance.

**Methods:**

14 patients with HCC were treated with the combination of cabozantinib and nivolumab through the Johns Hopkins Sidney Kimmel Comprehensive Cancer Center. Among them, 12 patients (5 responders + 7 non-responders) underwent successful margin negative resection and are subjects to tissue microarray (TMA) construction containing 37 representative tumor region cores. Using the TMAs, we performed imaging mass cytometry (IMC) with a panel of 27-cell lineage and functional markers. All multiplexed images were then segmented to generate a single-cell dataset that enables (1) tumor-immune compartment analysis and (2) cell community analysis based on graph-embedding methodology. Results from these hierarchies are merged into response-associated biological process patterns.

**Results:**

Image processing on 37 multiplexed-images discriminated 59,453 cells and was then clustered into 17 cell types. Compartment analysis showed that at immune-tumor boundaries from NR, PD-L1 level on tumor cells is significantly higher than remote regions; however, Granzyme B expression shows the opposite pattern. We also identify that the close proximity of CD8^+^ T cells to arginase 1^hi^ (Arg1^hi^) macrophages, rather than CD4^+^ T cells, is a salient feature of the TME in non-responders. Furthermore, cell community analysis extracted 8 types of cell-cell interaction networks termed cellular communities (CCs). We observed that in non-responders, macrophage-enriched CC (MCC) and lymphocyte-enriched CC (LCC) strongly communicate with tumor CC, whereas in responders, such communications were undermined by the engagement between MCC and LCC.

**Conclusion:**

These results demonstrate the feasibility of a novel application of multiplexed image analysis that is broadly applicable to quantitative analysis of pathology specimens in immuno-oncology and provides further evidence that CD163^-^Arg1^hi^ macrophages may be a therapeutic target in HCC. The results also provide critical information for the development of mechanistic quantitative systems pharmacology models aimed at predicting outcomes of clinical trials.

## Introduction

Primary liver cancers are predicted to be the third leading cause of cancer death in the US by 2040, and hepatocellular carcinoma (HCC) is the most common type of primary liver cancer (1, 2). The treatment of advanced HCC has benefited from the approval of multiple novel therapeutic agents, including anti-VEGF targeted agents (sorafenib, regorafenib, lenvatinib, cabozantinib, ramucirumab, bevacizumab) and immune checkpoint inhibitors (nivolumab, pembrolizumab, atezolizumab, and ipilimumab). In 2020, the combination of an anti-VEGF therapy (bevacizumab) plus the PD-L1 inhibitor atezolizumab was established as a new standard of care for patients with advanced HCC based on the demonstration of superior overall survival versus the prior standard of care sorafenib, and multiple additional combinations of anti-VEGF and immune checkpoint therapeutic combinations are in late stages of development. Despite these advancements, the overall survival of patients with HCC continues to lag many other tumor types, the majority of patients do not respond to current systemic therapies, and responses that are observed are usually transient.

We previously conducted a clinical trial of cabozantinib plus nivolumab in the neoadjuvant setting (3). Neoadjuvant trials present a unique opportunity to decipher the mechanisms of response and resistance to systemic therapies, by providing large tumor specimens for highly detailed spatial analysis of the TME that would not be possible with small biopsy specimens usually obtained in trials of advanced stage disease. Cabozantinib is a multityrosine kinase inhibitor (mTKI) of VEGFR2, c‐Met, Ret, Tie2, and AXL that is approved for patients with advanced stage HCC and is under investigation in combination with immune checkpoint therapy in HCC as well as other tumor types. Nivolumab is an inhibitor of PD-1. Patients with borderline resectable or locally advanced HCC were treated with 8 weeks of cabozantinib plus nivolumab, followed by surgical resection at week 12. Of 15 patients enrolled, 12 patients achieved surgical resection, and of these 12 patients who underwent resection there were 5 patients with major or complete pathologic responses. We subsequently performed multiplexed imaging analysis using Imaging Mass Cytometry™ (IMC) on 37 tumor region cores from responding and nonresponding resection specimens to understand mechanisms of response to this combination (3). An initial analysis of these specimens indicated that cabozantinib and nivolumab promote T cell-mediated antitumor immunity locally and systemically. Specifically, the unique aggregation of B cells was a hallmark of response with findings suggestive of their roles in antibody and pro-inflammatory cytokine production to indirectly support the antitumor immune response. The proximity of B and T cells to Ki-67^hi^ and PD-L1^hi^ macrophages was also a key characteristic of response. In tumor specimens from non-responders, however, the presence of arginase-1 expressing macrophages adjacent to B and T cells is associated with lack of response.

These findings revealed first-order tumor microenvironmental differences altered by the therapy. Tumor microenvironment (TME) is highly organized with spatially nuanced interactions between residing components. However, the characterization of this coordinated behavior remains relatively limited. To provide more integrated, deeper insights into such behavior, we performed multi-scale spatial analysis of the previously reported IMC data from our neoadjuvant cabozantinib and nivolumab trial to systemically interrogate the TME to identify other novel features of response and resistance to therapy and to quantitatively characterize the tumor immune microenvironment for subsequent applications to quantitative systems pharmacology (QSP). The analysis framework comprises the quantification of intra-tumoral phenotypic heterogeneity, stratification of tissue architectures, multi-cellular protein expression analysis, and network analysis of cellular communities. The results from these hierarchies were then summarized as a communication landscape in responders and non-responders, thus providing possible rationales for therapy response or resistance and guidelines for future treatment strategies. The proposed framework represents a novel application of multiplexed imaging in translational medicine to empower mining and correlating various microenvironmental distinctions to address cancer immunology questions. Importantly, the value of the framework also lies in its potential to parameterize and validate computational immuno-oncology models (4, 5).

## Methods

### Study Design

The study was designed to deeply interrogate the tumor microenvironment in pathologic non-responders versus responders to neoadjuvant cabozantinib and nivolumab treatment. Complete description of the trial and patient cohort, including patient demographical and clinicopathological descriptions can be found in our study (3). Briefly, this study was conducted at the Johns Hopkins School of Medicine. All cases (N = 15) were reviewed at the Johns Hopkins Sidney Kimmel Comprehensive Cancer Center Liver Cancer Multidisciplinary Clinic. Baseline demographic and disease characteristics are shown in **Table S1**. An oral dose of 40 mg per day of cabozantinib was administered to eligible patients for a total of 8 weeks. Patients received concurrent nivolumab at a dose of 240 mg IV every two weeks, for a total of four treatment doses following a two-week lead-in of cabozantinib monotherapy. Treatment-related adverse events are shown in **Table S2**. Patients received a restaging scan and surgical evaluation two weeks after their completion of neoadjuvant therapy. Patients determined to be eligible for surgical resection (evaluable N = 12) were subjected to a definitive surgical resection that was scheduled at least 28 days after the last dose of cabozantinib therapy, to reduce the likelihood of bleeding from cabozantinib and were subjected to downstream computational analysis. Figure legends contain information on sample sizes, experimental replicates, and statistical tests used.

### Data Acquisition With Imaging Mass Cytometry

37 representative tumor region cores of the 12 post-treatment FFPE surgical samples, each with a diameter of 0.6 mm, were subjected to the construction of tissue microarray (TMA). For one of the samples, 4 representative cores were selected, and cores were selected in triplicate for each of the remaining samples (n = 11). Controls cores (a normal tonsil and liver cores from a de-identified reference tissue archive at the Johns Hopkins Oncology Tissue Services) were also included in the TMA construction but were excluded from the computational analysis. The TMA was processed, covering dewax, antigen retrieval, and DNA labeling, using the protocol described elsewhere (3). Images were then acquired using a Hyperion Imaging System (Fluidigm, South San Francisco, CA).

### Image Processing and Analysis

The details of image segmentation were described elsewhere (3). Briefly, for each core, images highlighting the nuclei channels (Ir191 and Ir193), and the plasma membranes based on the IMC Cell Segmentation Kit (Fluidigm) were rendered and exported using MCD™ Viewer (Fluidigm). Next, a pixel classification algorithm (Ilastik) was applied to those images to generate the nuclei probability maps and the plasma membrane images. The resulting objects were converted to single-cell masks using CellProfiler v.3.1.8.1 to identify primary and secondary objects. To reduce the noise-to-signal ratio, all images for every channel were processed by automated LUTenhancement using ImageJ (NIH). The single-cell masks were then overlaid onto the cores that making it possible for the computation of cell-level spatial parameters and marker expression intensities of the cell markers. Finally, cell events were gated using FlowJo™ (BD) using a biaxial plot for Histone H3 *vs.* Ir191 intensities to eliminate artifacts related to antibody aggregates. The resulting single cells (n=59,453) were then clustered into metaclusters using FlowSOM 5 (6), which were then annotated into final cell phenotypes.

### Spatial Heterogeneity

A spatial form of Shannon’s entropy measuring the mixing level of a series of given set of cell types was computed for each core. The metric is defined as:


$$E_{SP}=-\sum_{i=1}^{n}\frac{d_{i}^{int}}{d_{i}^{ext}}p_{i}{\text{log}}_{2}p_{i}$$

where $d_{i}^{\text{int}}$ denotes the average Euclidean distance between all cells of type *i*; $d_{i}^{\text{ext}}$ represents the average Euclidean distance between all cells of set *i* and cells of all other types; *p_i_* is the percentage of type *i* within the core.

Cell-cell Euclidean distance was calculated using function ‘nn2’ from R package ‘flexclust’. Voronoi tessellations were generated using function ‘voronoi_polygon’ from R package ‘ggvoronoi’.

### Spatial Architecture

Visual inspection of Voronoi graphs (**Figures S2**, **3**) revealed three types of organizations: *compartmentalized, immune hot*, and *immune cold.* Such findings were corroborated using a marker-based cell-cell proximity metric (7). In brief, a cell is considered positive for a marker if the transformed expression intensity is larger than 0.5. For each given marker combination X and Y available in the panel list, the number of Y^+^ cells within 20 *μ*m of each X^+^ cell and their summation are computed as close interactions (denoted as *N*). To test whether *N* is significant, we fixed the locations of X^+^ cells and randomly permuted the locations of Y^+^ cells 500 times while keeping its overall density constant. The close neighbor counts were computed for each time to give a null distribution and the deviation of *N* from the null distribution was assessed using z-score, defined as:


$$z=\frac{N-\mu }{\sigma }$$

where *μ* and *σ* are the mean and standard deviation of the null distribution. For each core, z-scores were stored in a pairwise interaction matrix and clustered into different organizations encompassing HCC/hepatocytes markers, immune markers, or their mixtures. Organizations were then formally defined using *E_SP_*. In this context, we focused on the general cell lineages and only immune cells plus HCC/hepatocytes were considered when computing the *E_SP_*. According to the equation *E_SP_* described previously, the number of cell types can scale the entropy value, therefore *immune hot* and *immune cold* cores tend to have lower *E_SP_* due to relatively unified cell types. In this study, cores with a *E_SP_* > 0.8 (N = 9) were defined as *compartmentalized.* The validity of the cut-off threshold was confirmed by visual inspections of the Voronoi tessellation results for each group (**Supplementary Figure S3**). Of note, the selection of threshold is case-specific and should be re-evaluated in other contexts. The rest of the cores (N = 28) were defined as *mixed* (including *immune hot* and *immune cold)*.

### Identification of the HCC/Hepatocytes-Immune Boundary

The borders between two compartments were identified using an open-source image analysis software QuPath (8). For each *compartmentalized* core, tiff-format images with CD45^+^ and Pan-Keratin^+^ layers were exported, marking immune cells and HCC/hepatocytes. In QuPath, an artificial neural network-based pixel classifier was trained to discriminate immune and HCC/hepatocytes compartments and compartment mask, whichever touched less core boundary, was exported as a JSON file containing a point set of border lines. The file was then cleaned (removal of all core boundary points) to yield the final border. The distance of a cell towards boundary was defined as its distance to the nearest boundary point. Cells were then defined as either *close* (< 40*μ*m), or *far* (≥ 40*μ*m) in *HCC/hepatocytes compartment* or *immune compartment*.

### Profiling of Cell Infiltrations

The profile was constructed for *compartmentalized* cores only. For each core, two histograms of distances of cells to boundary (bin width = 20*μ*m), one for each compartment, were generated. The histogram reflected the number of encompassed cells with respect to each distance bin and the numbers were further broken down (sub-histograms) based on cell types. To distinguish *immune* and *HCC/hepatocytes compartments*, we mirrored the sub-histograms for *HCC/hepatocytes compartment* to the left half axis. Each bar of sub-histograms was then transformed to a single point marking the cell counts and connected within its same group to form consecutive profiles. Cell counts were normalized by the corresponding boundary length to make profiles comparable across cores.

### CD8^+^ T Cell RiskScore

For each CD8^+^ T cell from cores with *mixed* architecture, we computed the Euclidean distance to its nearest CD4^+^ T cell (denoted as *d*1) and nearest hazard macrophages (*d*2). The score was then defined as:


$$\frac{d1}{d1+d2}$$

### Identification of Cell Community (CC)

To identify CCs, Delaunay triangulations were computed such that cells within 20*μ*m were connected. CCs were defined as closed networks containing at least 10 cells, with each node representing the centroid of a cell (labeled by corresponding cell types) and edges representing interactions between nodes. The process was carried out for cores, and for each detected network, the number of nodes of different labels was stored in the network component matrix. The matrix was subjected to hierarchical clustering to group communities with similar components.

### Communication Analysis

Networks detected as previously described were transformed to fixed-length feature vectors using an unsupervised graph embedding framework *graph2vec* (9). In brief, rooted sub-graphs were generated by negatively sampling and relabeling the nodes. Then, a skip-gram model was trained to optimize the probability of predicting subgraphs that exist in the given network and embedding was iteratively learned over several epochs. Considering the low scale of each network, the embedding size was chosen as 16. For each core, Spearman’s rank correlation test was performed for each pair of graph embedded vectors. Since each network also belongs to a CC, correlation test between two networks was regarded as one trial test between two CCs. For each pair of CCs, the fraction of significant trials (Wilcoxon rank-sum test *p* < 0.05) was computed as the communication strength between the corresponding CCs. This procedure was conducted for responders and non-responders separately. For each case, a communication network was mapped with each node representing one CC type and darkness of edges representing the communication strengths between connected ends (blank indicates no communication and dark indicates strong communication). CC types that solely belong to one patient were excluded from the analysis.

### Statistical Analysis

Two-sided Wilcoxon rank-sum test was performed for pairwise comparisons. FDR-adjusted p < 0.05 was considered significant. As previously described (10), exclusion test on *RiskScore* was implemented by excluding data from each core/patient iteratively. For each iteration, *RiskScore* between responders and non-responders was modeled using a linear-mixed effects model that treating each core/patient identifier as a random effect. The p-values were computed using Satterthwaite’s degrees of freedom method. The method was implemented using ‘lmer’ function from R package ‘lme4’ (11). Heatmaps with hierarchical clustering (Metric: One minus Pearson correlation; Linkage method: Average) were generated using Morpheus software (https://software.broadinstitute.org/morpheus).

## Results

### Patient Characteristics

From April 2018 until September 2019, we enrolled 15 patients through the Liver Cancer Multidisciplinary Clinic at the Johns Hopkins Sidney Kimmel Comprehensive Cancer Center in Baltimore, MD. The study is fully described in our previous work (3). Of these patients, 12 patients underwent successful margin-negative surgical resections of their tumors. Of the 12 patients, 4 patients had major pathologic responses (at least 90% tumor necrosis) and 1 patient had a complete pathologic response. In the following context, these 5 patients are referred to as responders (R) and the remaining 7 patients are referred to as non-responders (NR).

### Image Mass Cytometry Enables Cellular Level Profiling of Tumor Microenvironment

**Figure 1:**
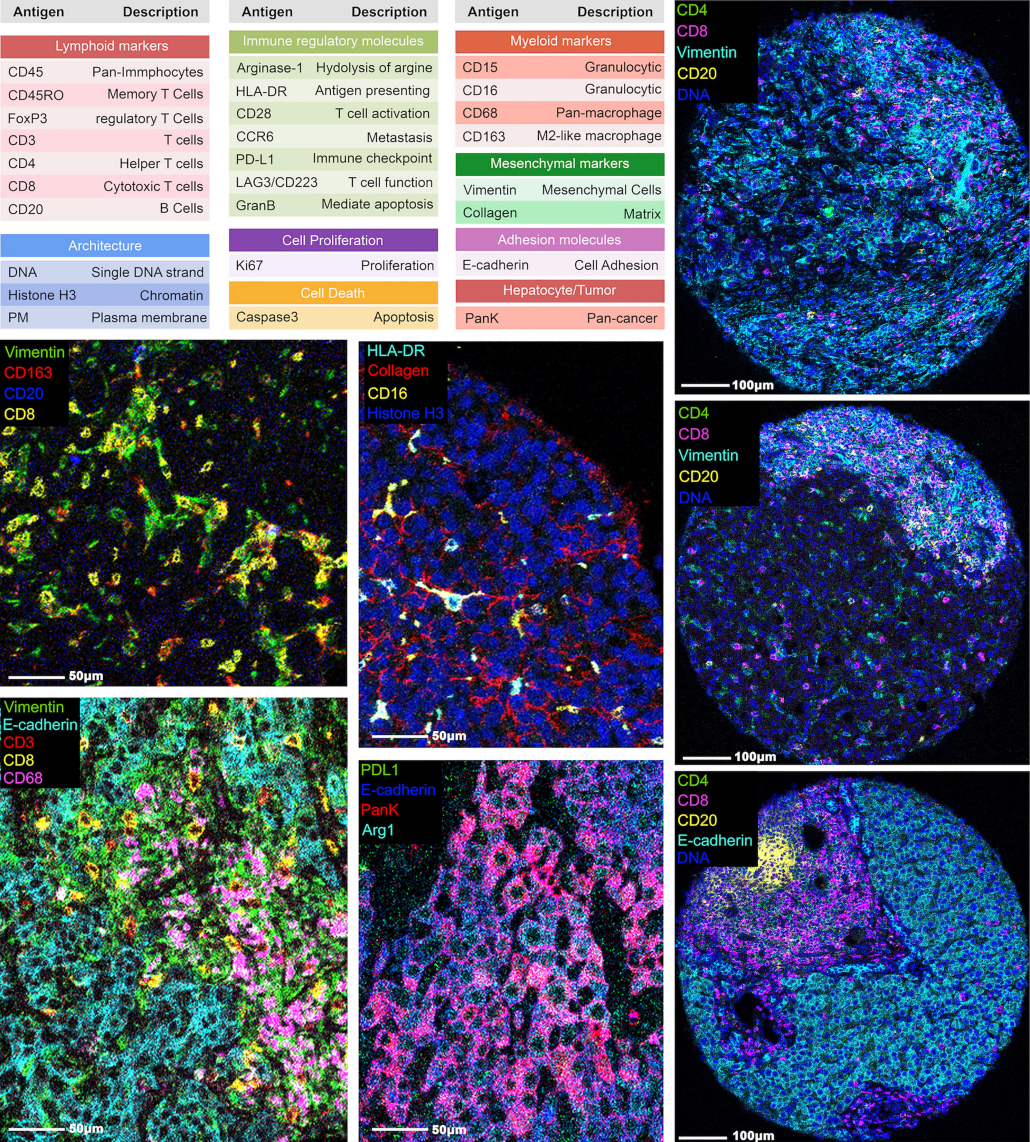
Representative images from IMC. A panel of 27 markers was used to stain the hepatocellular carcinoma tumor region cores and processed using IMC. The marker names and descriptions are included.

**Figure 2:**
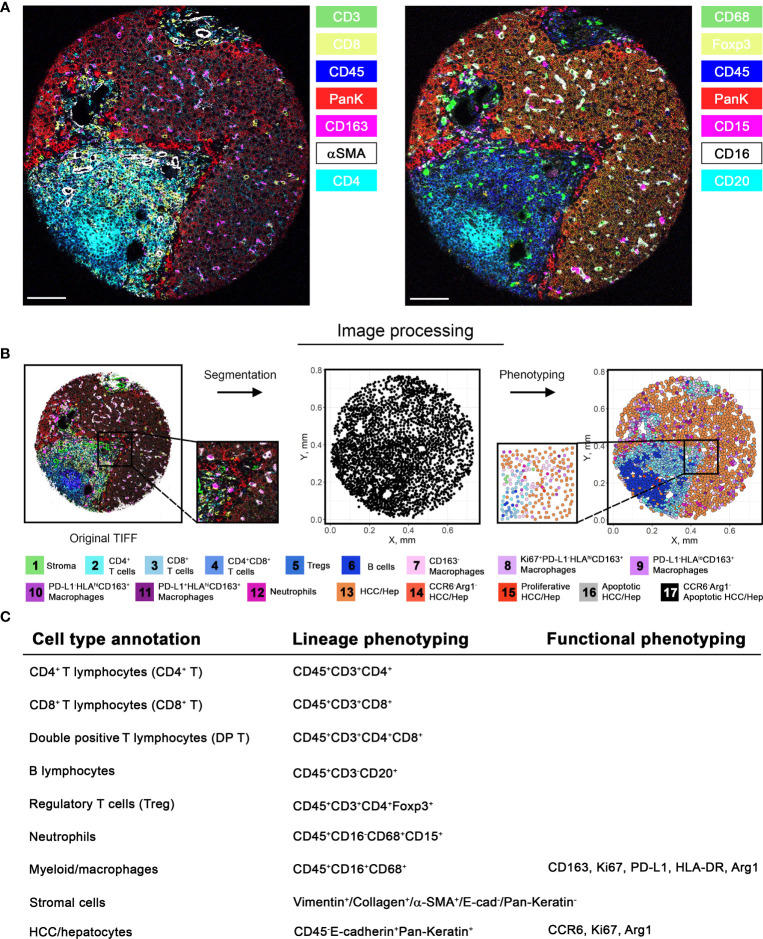
Image processing pipeline and cell phenotyping. **(A)** Among 27-marker panel stained in this study, 14 markers were selected and visualized separately as 7-channel multiplexed images capturing different subsets of cells. **(B)** Image processing pipeline was initiated by image segmentation based on nucleus and plasma membrane markers. Segmentation allows extraction of marker intensities for each cell, which enables clustering of marker combinations. Clusters were then annotated into final cell phenotypes (unannotatable/non-cell cluster is not included). Markers used to further subtype the clusters are described in **(C)**. Note that the final annotations are composed of both lineage and additional phenotyping markers.

Using the 12 surgically resected tumor samples, we constructed a tissue microarray in triplicate from 11 samples and in quadruplicate from 1 sample, yielding 37 representative tumor region cores. Using the tissue microarray, we performed image mass cytometry (IMC) with a panel of 27-markers (**Figure 1**). The resulting multiplexed images were segmented into a dataset of single-cell descriptions using a previously established image processing pipeline. In total, segmentations discriminated 59,453 cells from 37 cores. FlowSOM algorithm further clustered these cells into 18 annotated phenotypes, including 1 stroma/architecture phenotype; 1 B cell phenotype; 1 CD4^+^ T cell phenotype; 1 CD8^+^ T cell phenotype; 1 double-positive cell phenotype; 1 regulatory T cell phenotype; 1 neutrophil (PD-L1^+^) cell phenotype; 5 myeloid/macrophage phenotypes; 5 HCC/hepatocyte phenotypes (**Figures 2A, B**); and 1 unannotatable/non-cell cluster (UA noncell). In the following context, UA noncell cluster was removed from calculations and further analyses and resulting in 58,740 evaluable cells in total. Their first-order distribution measurements (densities) were summarized in **Table S3**. The markers used to annotate cell clusters are listed in **Figure 2C**. Technical details and clustering results from FlowSOM algorithm were described in our previous study (3, 6).

**Figure 3:**
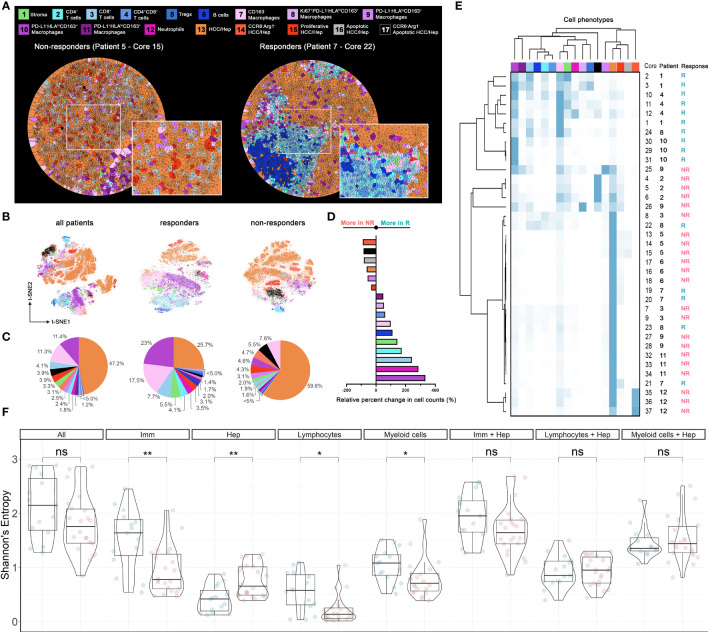
Cell populational characteristics and heterogeneity. **(A)** Voronoi tessellation results for exemplar responder and non-responder cores. Each partitioned polygon was color-coded by corresponding cell type. Cell distributions evaluated using **(B)** t-SNE visualization (n = 58,740), **(C)** pie-chart, **(D)** waterfall, and **(E)** heatmap and hierarchical clustering. **(F)** Spatial Shannon’s entropy computed for different sets of cell types. Significant differences were observed for immune cell subset, tumor cell subset, lymphocytes subset, and myeloid cell subsets between responder samples (n = 15) and non-responder samples (n = 22). P values were computed using Wilcox rank-sum test and adjusted using FDR. *p value < 0.05; **p value < 0.01. ns, not significant.

To explore the overall spatial distribution pattern associated with specific cell phenotypes, we performed Voronoi tessellation for each core such that individual cells were allocated to polygons based on proximity to surrounding cells. Visual inspection of tessellated results from responders shows tight packing with immune cells whereas tumor cells are predominantly present in cores from non-responders (**Figure 3A** and **Supplementary Figures S2**, **S3**). Similar patterns are confirmed by t-SNE plots based on the normalized expression matrix with respect to all cores, and cores from responders and non-responders (**Figure 3B**). Quantifications reporting the percentage of each cell type further corroborated such tendency (**Figure 3C**). In general, the number of tumor cells (~58.1%) is comparable to that of immune cells (~40%). However, cores from responders are dense with immune cells (~64.5%) and cores from non-responders are dense with tumor cells (~76.2%). We then computed the relative change in each cell phenotype of non-responders compared to responders: neutrophils, CD163^+^PD-L1^-^HLA^hi^ macrophages, CD163^-^ macrophages, stromal cells, plus all lymphocytes and all macrophages (except CD163^+^PD-L1^-^HLADR^lo^ subtype) were prevalent in R, whereas all tumor cells and CD163^+^PD-L1^-^HLADR^lo^ macrophages were prevalent in NR (**Figure 3D**). Such observations are consistent with previous finding that an immuno-activated microenvironment may favor response to immune checkpoint therapy (12). In-depth profiling of cell compositions based on hierarchical clustering revealed that none of NR-associated cores were dense with lymphocytes, whereas R-associated cores could have high portion of tumor cells. Within each response group, there were clear patient-specific differences; in addition, even though cores from the same patient tended to be similar, there were observable differences (**Figure 3E**). These data highlight the intra- and inter-tumoral heterogeneity across the studied cohort, which may account for some of the variability in patient response to immunotherapy (13). To directly compare the region-specific spatial heterogeneity, a spatially adjusted Shannon’s entropy (*E_sp_*) was computed for each core. In brief, the metric takes the Euclidean distance between spatial objects into consideration (14); an increase in distances between cells of the same phenotype and a decrease in distances between cells of different types will collectively lead to the increase of *E_sp_* (see *Methods*). The computation was conducted 8 times, each with a different combination of cell phenotypes (**Figure 3F**). Results showed that the entropy levels when all cell types were considered were not distinguishable between R and NR, however, R-cores were found to be more heterogeneous in terms of immune cells (FDR-adjusted *p* < 0.01), lymphocytes alone (FDR-adjusted *p* < 0.05), and macrophages alone (FDR-adjusted *p* < 0.05); and NR-cores were found to be more heterogeneous in terms of tumor cells (FDR-adjusted *p* < 0.01). Such findings suggested that tumor-infiltrating immune cells of different types co-exist in the R-cores, whereas NR-cores were less infiltrated by immune cells.

### Intercellular Protein Interactions Characterize Spatial Architectures in HCC Tumor Microenvironment

**Figure 4:**
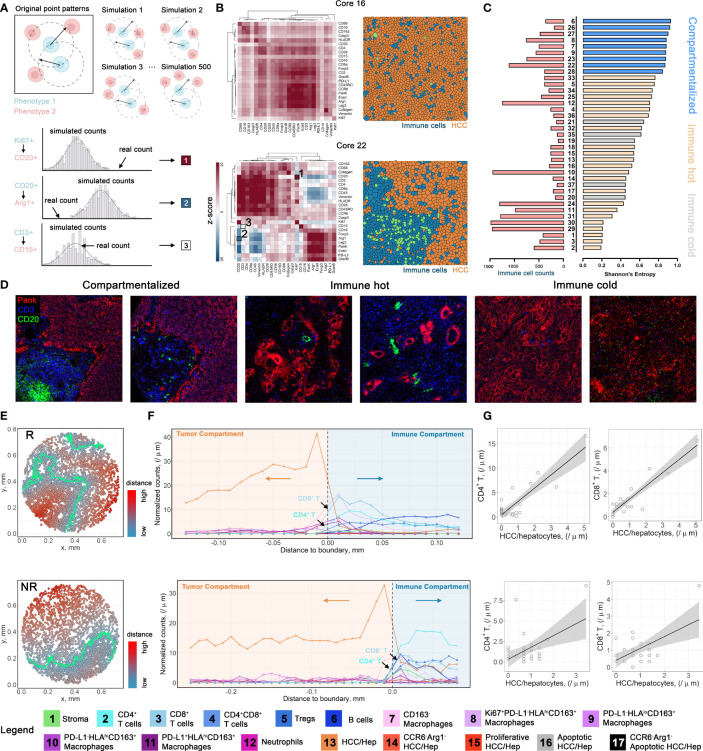
Spatial architecture characterization and cellular infiltration profiling. **(A)** Multicellular protein-protein interaction methodology diagram. **(B)** Positive z-score indicates spatial clustering (red); negative z-score indicates spatial separation (blue); ~0 z-score indicates no spatial correlations (white). **(C)** Corresponding cores from the first quartile of Shannon’s entropy were defined as *compartmentalized* cores. Remaining cores with immune cell count > 200 were defined as *immune hot*, otherwise as *immune cold*. **(D)** Image snippets for different tissue structure types. **(E).** Point patterns describing the relative distances to HCC/hepatocytes-immune boundaries for core 22 (upper) and core 8 (lower). Cells are color-coded by their distances to HCC/hepatocytes-immune border. **(F).** Infiltration profiles of each cell type from cores highlighted in **(E)**. **(G)** Exemplar correlations between normalized cell counts of different cell types. Correlations were performed using Pearson’s method with FDR-adjustment for p-values.

To further assess the spatial heterogeneity hierarchy at the cellular level, we employed a previously described method (see *Methods*) to quantify the spatial interactions for pairs of markers that contribute to the differential tissue structures across cores with varying cell components and abundance (7). In brief, for a given pair of proteins (X and Y), we first computed the number of Y-positive cells (Y^+^) within 20*μ*m of X^+^ cells and defined as *neighbor counts*. Next, we repeatedly permuted the locations of Y^+^ cells to formulate a null distribution of *neighbor counts* and a z-score was calculated to reflect the overall spatial proximity between the given proteins (**Figure 4A** and **Supplementary Figure S4**). In general, high z-scores indicate spatial clustering and conversely low z-scores indicate spatial dispersion. We applied the metric across all cores and clustered the resulting z-scores. Collectively, we observed two prototypical tissue architectures: (i) colocalization of tumor and immune cells, demonstrated by heatmaps with overall high z-scores for most of the immune and tumor-associated markers; and (ii) compartmentalization, demonstrated by heatmaps with high z-scores within, and low z-scores across tumor- and immune-exclusive markers (**Figure 4B**). To formally discriminate compartmentalized cores, we ranked the ***E_sp_*** and the cores from the third quartile were defined as *compartmentalized* cores. Remaining cores were then defined as *mixed* cores and further classified to *immune hot* (immune cell counts ≥ 200) and *immune cold* (**Figures 4C, D**). This criterion coincided with our observations that *mixed* cores were generally dominated by either immune cells or HCC/hepatocytes, therefore more uniform in terms of cell phenotypes; whereas *compartmentalized* cores entailed comparable amounts of both cell types, therefore conferred an abundant heterotypic cell-cell interactions. This process resulted in 8 *compartmentalized* cores (2 from R and 6 from NR), 24 *immune hot* cores (10 from R and 14 from NR), and 5 *immune cold* cores (3 from R and 2 from NR). For *compartmentalized cores*, we then used a supervised artificial neural network algorithm (see *Methods*) to generate tumor and immune masks, and thus the borderlines separating the compartments (**Figure 4E**). To gauge the biological differences between compartments, we first constructed cellular infiltration profiles for compartmentalized core by computing the distances toward HCC/hepatocytes-immune borders for all cells and split to each specific cell type (**Figure 4F** and **Supplementary Figure S5**). Next, we correlated normalized cell counts for each immune cell phenotype to the HCC/hepatocytes. Results revealed significant correlations between cell types: for instance, in core 22 (responders), strong positive spatial correlations between CD4^+^ T cells (FDR-adjusted *p* = 2.9845e^-7^), CD8^+^ T cells (FDR-adjusted *p* = 1.0015e^-8^) and HCC/hepatocytes infiltrations, which may confer an activated antitumor immunity (**Figure 4G** and **Table S4**). However, correlations were inconsistent within response groups, therefore hampers the establishment of biomarkers at cell distribution level.

### Spatial Quantifications of Multi-Cellular Protein Expressions Reveal Ties to Therapeutic Response

**Figure 5:**
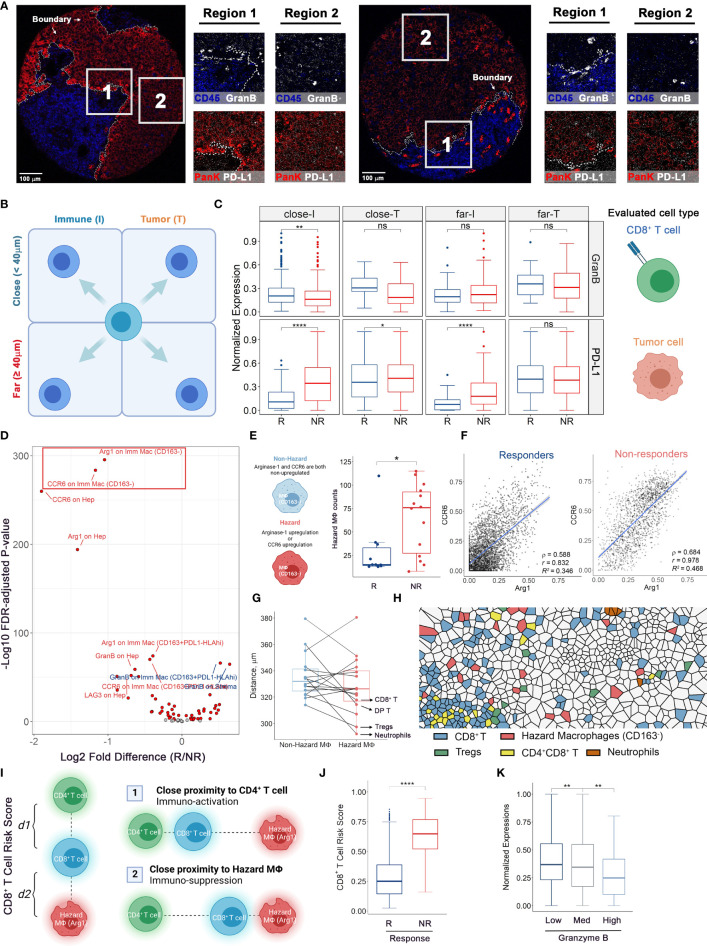
Multicellular protein expressions at HCC/hepatocytes-immune border reveal associations to immune-therapy outcomes. **(A)** Color overlays of lineage proteins covering Pan-Keratin and CD45 (red and blue) and functional markers covering PD-L1 and Granzyme B (white) in whole tissue core and subregions. **(B, C)** Protein expression analytical strategy. For compartmentalized cores (n = 2 from R and n = 6 from NR), functional marker expressions on target cells were examined adjacent and remote to HCC/hepatocytes-immune border and truncated to treatment response criteria for comparisons. **(D)** Volcano plot showing the comparison results of marker intensities on various cell types in *mixed* cores (n = 10 from R and n = 8 from NR, same for the rest legends). Red dots: FDR-adjusted P values < 0.01. Text colors indicate whether the mean marker expression was higher in non-responders (red) or responders (blue). **(E)** Hazard macrophage was defined such that at least one protein of CCR6 and Arg1 was upregulated on CD163^-^ macrophages. **(F)** Correlation plots showing the co-expression pattern of CCR6 and Arg1 on CD163-negative macrophages. **(G)** Quantifications of shortest distances between two macrophage subtypes and other cell types. **(H)** Voronoi tessellation map highlighted for hazard-, non-hazard macrophages and B cells. **(I)** Diagram of CD8^+^ T cell *RiskScore*. Denote each CD8^+^ T cell to its nearest hazard macrophages as *d1* and to its nearest CD4^+^ T cell as *d2*, thus the *RiskScore* is formally computed by taking the proportion of *d2* to the combined distance of *d1* and *d2*. **(J)** *RiskScore* on per-cell basis for responders (evaluable n = 8) and non-responders (evaluable n = 12). **(K)** Normalized Granzyme B expressions on per-CD8^+^ T cell basis for low-, medium-, and high-*RiskScore* groups. P values were computed by two-tailed Wilcoxon’s rank-sum test with adjustment for multiple comparisons (FDR). *p value < 0.05, **p value < 0.01, ****p value < 0.0001. ns, not significant.

To derive deeper biological insights from the different spatial architectures, we first gauged the expression patterns of two key markers: programmed death-ligand 1 (PD-L1), an immune-regulatory checkpoint molecule, and Granzyme B (GranB), an antitumor effector molecule, in compartmentalized cores (**Figure 5A** and **Supplementary Figure S6**). We defined regions less than 40μm from the HCC/hepatocytes (T) - immune (I) border as “close” and otherwise as “far” and further categorized them into close-I, far-I, close-T, and far-T regions based on their associated compartment. We then collected normalized expression values of aforementioned markers and compared them among the four regions **(** **Figure 5B** **)**. Based on known biology, GranB was evaluated on CD8^+^ T cells and PD-L1 was evaluated on HCC/hepatocytes **(** **Figure 5C** **)**. Striking differences between responders and non-responders were detected for GranB expressions in CD8^+^ T cells only in close-I (FDR-adjusted p = 0.00123) and in PD-L1 expressions in HCC/hepatocytes in close-I and far-I regions (FDR-adjusted p < 10^-4^), but not for other regions. These results showed that the spatial relationships between CD8^+^ T cells and HCC/hepatocytes with respect to the HCC/hepatocytes-immune border are associated distinctly with response. We then evaluated the marker expression patterns in cores with mixed architectures and immune cell counts greater than 200. We compared the expression levels of all evaluable functional markers (Arginase-1 [Arg1], CCR6, CD28, GranB, HLA-DR, Ki67, LAG-3, PD-L1) on various cell types. Interestingly, we observed that the expression of Arg1 and CCR6 was drastically upregulated on CD163^-^ macrophages in non-responders **(** **Figure 5D** **)**. Based on this observation, we were able to further define two CD163^-^ macrophage subtypes based on the co-expression pattern of Arg1 and CCR6: non-hazard macrophage (both signals were relatively low) and hazard macrophage (at least one signal was upregulated) (**Supplementary Figure S7A**). Not surprisingly, hazard macrophages were found to be predominantly associated with non-responders (**Figure 5E**). Pairwise correlations displayed a high linearity between the two markers in general (**Figure 5F**). Interestingly, we found that the mean distances of hazard macrophages to CD8^+^ T cells, double positive (CD4^+^CD8^+^) T cells, regulatory T cells (Tregs), and neutrophils all decreased **(** **Figure 5G** **)** and visual inspections of Voronoi tessellations also confirmed such finding (**Figure 5H** and **Supplementary Figure S7B**). These were all major cell types expressing CCR6. Therefore, the upregulation of CCR6 can be achieved through engagement of CCR6-macrophage inflammatory protein 3α (MIP-3α) signaling axis adjacent to hazard macrophages. Considering the immunosuppressive role of Arg1^+^ has been well established (15, 16), we selected a subset of hazard macrophages (Arg1^hi^) to further explore their biological significance. Over the years, the geographical proximity between immune components has been increasingly explored (17–19). In this study, we proposed CD8^+^ T cell RiskScore – a modified version of SpatialScore (20), to represent the relative distance of CD8^+^ T cell to CD4^+^ T cell and hazard macrophage. Of note, SpatialScore evaluates the behavior of CD4^+^ T cells, rather than CD8^+^ T cells. Hence, modifications are made to the marginal cell types to recapitulate the proxy of the balance between effector T cell activity and suppression, as described by SpatialScore. Scores were computed on a scale of 0 to 1, with lower values representing the CD8^+^ T cells that are located in the vicinity of CD4^+^ T cells and higher values representing the CD8+ T cells are located in the vicinity of hazard macrophages (see *Methods)*. CD8^+^ T cells in the vicinity of CD4^+^ T cells are more likely to be further helped in their antitumor roles, whereas those near hazard macrophages may be suppressed (**Figure 5I**). Upon computing the scores for each CD8^+^ T cell from cores with *mixed* architectures (evaluable n = 20 as 4 cores were removed for lack of either cell type), we observed that the computed *RiskScore* in non-responders was significantly elevated (Wilcoxon rank-sum test *p* = 7.65e^-65^), suggesting that the macrophage-mediated inhibition of CD8^+^ T cell may contribute to the lack of response (**Figure 5J** and **Supplementary Figure S7C**). Given the modest sample size, we further tested whether such significance was biased that originated from a single core/patient. Thus, we further performed exclusion analysis on the *RiskScore* data. At core-level, we iteratively excluded one of the 20 cores. At patient-level, we iteratively excluded cores from the same patient (evaluable N = 10). For each iteration, *RiskScore* between responders and non-responders were modeled using a linear-mixed effects model that treating each core/patient identifiers as a random effect and p-values were computed using Satterthwaite’s degrees of freedom and visualized as dot plots (*Methods*). Results showed that the statistical significances were retained over all iterations under both conditions, therefore confirming the robustness of *RiskScore* (**Figures S7D, E** **)**.

To further ascertain the functional state of CD8^+^ T cells among the different risk scores, we attributed CD8^+^ T cells to three groups: (i) low-risk, with scores lower than 0.3; (ii) medium-risk, with scores between 0.3 and 0.7; (iii) high-risk, with scores greater than 0.7. The thresholds were selected to split the whole interval into approximate thirds (**Supplementary Figure S7E**). We then compared the expressions of GranB among the risk groups, finding that the GranB expressions were significantly higher in low-risk group (FDR-adjusted *p* = 0.004) and lower in high-risk group (FDR-adjusted Wilcoxon rank-sum test *p* = 0.003), compared to the medium group (**Figure 5K** and **Supplementary Figure S7F**). This demonstrated that greater proximity to CD4^+^ T cells associates with higher GranB on adjacent CD8^+^ T cells (low *RiskScores*), whereas greater proximity to the hazard macrophages associates with lower expression of GranB. These findings suggest that the lower expression of cytotoxic molecules (e.g., GranB), in the context of the close proximity to hazard macrophage and greater distance from CD4^+^ T cells, translates into resistance to anti-PD1 immunotherapeutic regimen.

### Network Analysis of Cellular Community Reveals Communication Landscape in HCC Tumor Microenvironment

**Figure 6:**
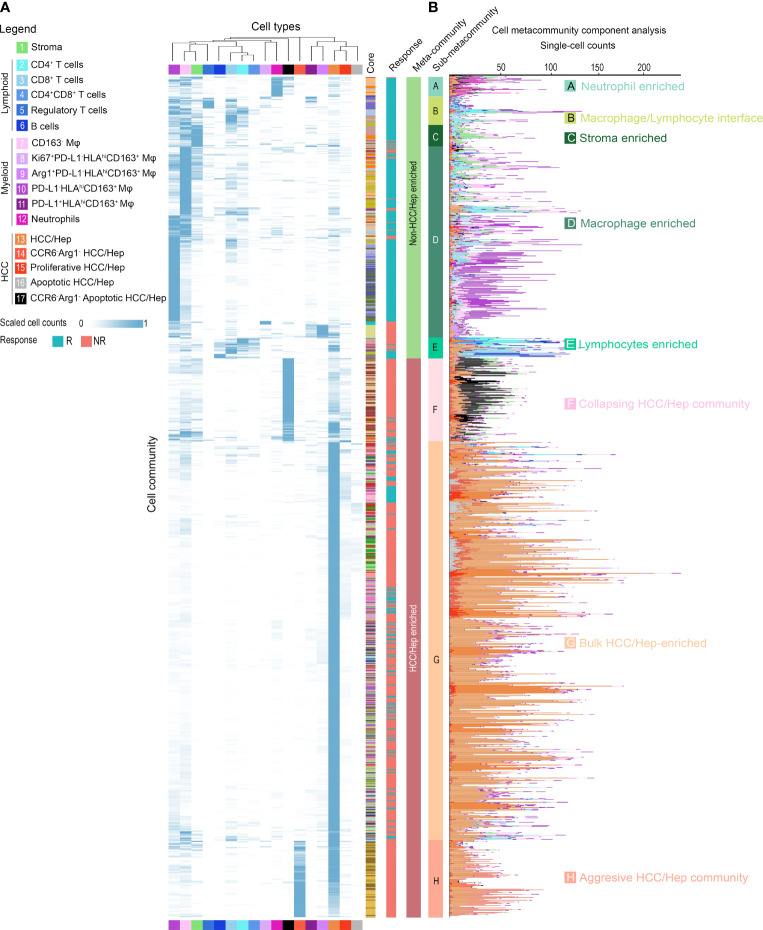
Cellular community analysis identifies cell communities within HCC tumor microenvironment. Hierarchical clustered **(A)** heatmap and **(B)** stacked bar plot of scaled and absolute cell-type counts in each cell community. Colored columns indicate the originated core (n = 37), response criteria, assigned community and meta-community type.

**Figure 7:**
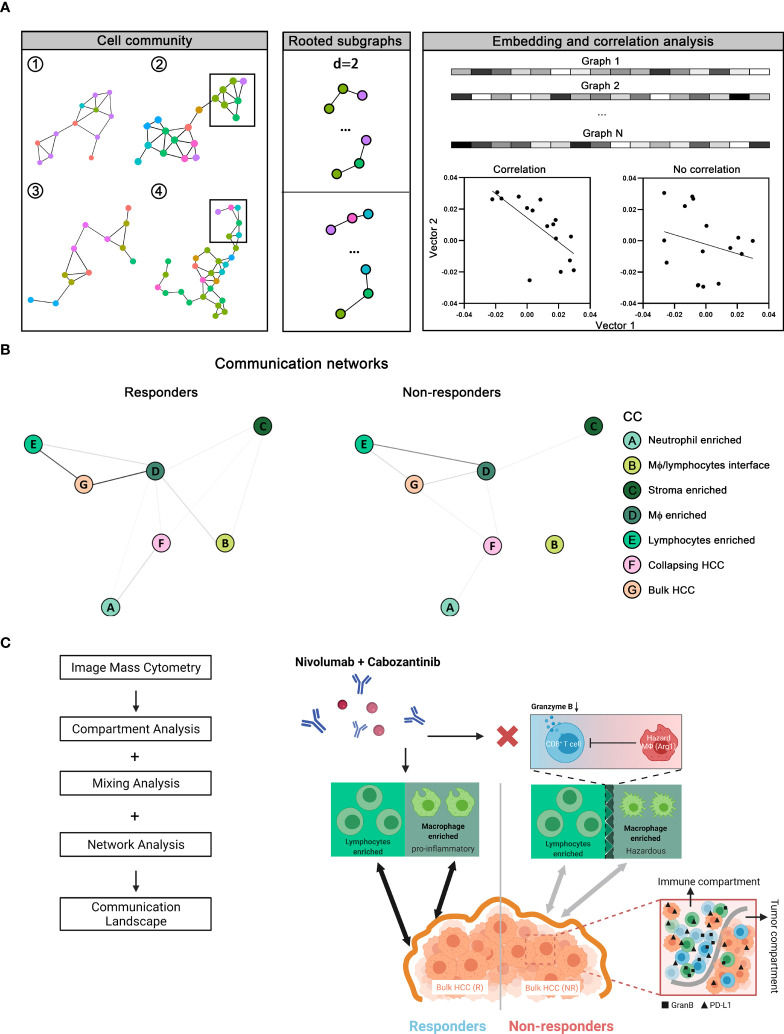
Network analysis of communication landscape modeling of the tumor microenvironment in responders versus non-responders. **(A)** Cellular communities (CCs) from all cores (n = 37) identified as previously described were converted to graph-embedded vectors. Correlation tests were then performed to assess the association between each given pair of vectors. **(B)** Correlation test results give rise to two distinct communication maps in tumor microenvironment associated with responders and non-responders. **(C)** Results from network analysis, together with compartment and mixing analysis, were summarized into communication landscape models that link to the therapy response.

Finally, since we anticipate the spatial analysis of single-cell interactions to inform population behavior shifts in the tumor microenvironment, we further extracted a series of cellular communities (CCs) - collections of ‘neighboring’ cells - and then grouped into 2 general meta-clusters using hierarchical clustering: non-HCC/hepatocytes versus HCC/hepatocytes (**Figure 6A** and **Supplementary Figure S8A**). While the clustering was performed based on the quantities of cell components, a reasonable separation of responsiveness was also noted. Meta-clusters were further discriminated into 8 types: (A) neutrophils-enriched; (B) macrophages/lymphocytes interface; (C) stroma-enriched; (D) macrophages-enriched; € lymphocytes-enriched; (F) collapsing HCC/hepatocytes (apoptotic HCC/hepatocytes)-enriched; (G) bulk HCC/hepatocytes-enriched; and (H) aggressive HCC/hepatocytes-enriched (**Figure 6B** and **Supplementary Figure S8B, C** **)** based on majority cell types. Of note, CC type H was seen only in cores from patient 12 and thus excluded from further analysis to avoid bias. Non-HCC/hepatocytes CCs predominantly existed in responders, conversely HCC/hepatocytes CCs were more abundant in non-responders (**Figure 6B** and **Supplementary Figure S8D**). To analyze their spatial communications, we converted each CC network to vector. The communications between CCs were then assessed using correlation test on paired CC vectors of the corresponding types (**Figure 7A**, see *Methods* for details). In brief, every CC (2-D object) was embedded into a vector (1-D object). For each pair of CCs residing within the same core, we computed the p-value using correlation test between the respective vectors. We then sorted the test results into sets based on the CC types and the communication level was quantified as the proportion of significant p-values in the corresponding set and summarized as correlation matrix, which was then converted into communication map. The formulated maps described the signaling landscape in responders and non-responders, with nodes associated with CC types and darkness of links associated with the communication strengths (**Figure 7B**). Special attention was given to the pattern that bulk HCC/hepatocytes-enriched CCs (node G) were strongly communicating with lymphocytes-enriched CCs (node E) and macrophages-enriched CCs (node D) in responders; however, such pattern was replaced by the communication between node E and node D in non-responders. This observation supports the previous findings at network level that hazard macrophages may impair the cytotoxicity of CD8^+^ T cells and promote the lack of response.

Taken together, our results have demonstrated distinct communications landscapes in the tumor microenvironment in responders versus non-responders (**Figure 7C**). In summary, based on our multi-scale analysis, (i) the synergistic anti-tumor immunity of macrophages and lymphocytes favors cabozantinib and nivolumab, (ii) immune function regulators (i.e., GranB and PD-L1) were upregulated throughout the immune compartment in non-responders, (iii) Arg1/CCR6-expressing macrophages (hazard macrophages) is also a prominent feature in non-responders, (iv) close proximity to Arg1^hi^ hazard macrophages and distance away from CD4^+^ T cells associate with poorer effector function of CD8^+^ T cells.

## Discussion

We used a highly integrative, multi-scale analytic framework to discover features of response to modern systemic therapy in HCC. Using imaging mass cytometry on 37 tumor cores representing different regions with a panel of 27 biomarkers, we extracted a single-cell database of 59,453 cells highlighting 4 cell lineages, marking stroma, lymphoid cells, myeloid cells, and HCC/hepatocytes, with different functional states, and marking expressions of PD-L1, Arginase-1 (Arg1), CCR6, Ki67, and CD163. As reported previously (3), demographic analysis on cell frequencies revealed that lymphocytes were more abundant in responder cores, and not surprisingly, HCC/hepatocytes were more likely to be seen in non-responder cores. Intra-tumoral heterogeneity (ITH) is a hallmark of therapy resistance, and a major source of ITH is phenotypic heterogeneity. Existence of immune and HCC/hepatocytes with diverse spatial gradients might constitute microenvironmental cues that trigger poor clinical outcomes (13, 21). To quantitatively assess phenotypic ITH, we computed spatial Shannon’s entropy on total cell populations and subsets, which reflects the integration of inputs from cell type diversity, counts diversity, and their spatial orientations. We showed that responder cores have higher entropy scores in immune subpopulations. This observation is therefore consistent with the observation that highly immune-inflamed TME (hot tumors) is associated with enhanced immune checkpoint blockade response (22). Using entropy score, we then categorized tumor region cores into two architectural models: *compartmentalized*, in which we observe clear separation of immune and HCC/hepatocytes; and *mixed*, in which mixtures were otherwise noticed. For *compartmentalized* architecture, we observed downregulated GranB expressions at HCC/hepatocytes-immune border in non-responders; and upregulated PD-L1 expressions throughout the immune compartment. Such associations are supported by previous studies that GranB^lo^ CD8^+^ T cells associated with reduced cytotoxicity and PD-L1^hi^ HCC/hepatocytes are highly prevalent in aggressive tumors – both may impair the control in tumor progression (23, 24). For *mixed* architecture, we identified a strong co-expression signal of Arg1 and CCR6 on CD163^-^ macrophages. The presence of CCR6 on CD163^-^ macrophages and its role in immune regulation remain unclear. Previous studies suggested that the co-localization of *MIP-3α* and *CCR6* could promote cancer cell invasion (25). Based on our current analysis, we hypothesize that the elevated expression of CCR6 on these macrophages may be linked to the functioning of CCR6/MIP-3*α* signaling axis in line with the observed association between CCR6^hi^ hazard macrophages and the lack of response to therapy. The immunosuppressive role of Arg1^hi^ myeloid cells has been extensively reviewed previously (15, 26–30). Furthermore, we introduced CD8^+^ T cell *RiskScore*, a quantitative measurement to evaluate the dysfunction risk of CD8^+^ T cell in HCC. We found that CD8^+^ T cells with high *RiskScore* were predominantly present in non-responders and found to have significantly lower GranB expression when compared to CD8^+^ T cells with medium or low *RiskScore*. These results highlighted the significance of distance relationships among immune cells in assessing immuno-therapy responses. Finally, we deconstructed all the spatial features into distinct cellular communication networks found in responder and non-responder TMEs. This approach illustrated that there are specific networks of cells within the TME that are highly coordinated and associated with response to immunotherapy. Again, in responders, cellular communities of macrophages and lymphocytes jointly characterized the tumor microenvironment of responders, whereas macrophage-mediated immunosuppressive communication networks were noted in non-responders. Our analytic approach in this study independently recapitulates the relationship between immunosuppressive myeloid cells and antitumor T cells that we have previously observed within the same dataset and again emphasizes the importance of working to target such immunosuppressive biology to advance the treatment strategies against HCC. In addition, the single-cell properties derived from the framework can be utilized to facilitate development and calibration of computational immuno-oncology models at spatial resolutions (31–33). Such three-dimensional models bridged the translation between 2D pathology biopsies and their 3D reconstruction that enabling a more precise mechanistic systems biology modeling of response to immunotherapy.

There are important limitations in our study. First, we utilized a relatively small cohort and future validation on a larger cohort is required. It is worth noting that in region-specific analyses, we were able to treat each core as a separate data point. Analyzing core-level data enables the analysis of intra-tumoral heterogeneity from a given patient, thus entailing a generalizable framework that is less sensitive to issues related to sampling bias at the level of individual patients. Second, an inherent analytical limitation originates from the set of markers available in the dataset. For example, the lack of programmed cell death protein 1 (PD-1) expression data in our dataset precluded our ability to evaluate the spatial characteristics of the PD-1/PD-L1 signaling axis. Besides, previous studies also revealed the activation of transcriptional factors such as STAT, NF-κB, and HIF-1*α* are hallmarks of HCC progression and metastasis, therefore including transcriptional factors in the IMC staining panel is warranted (34–36). In addition, when interpreting T cell interactions, including the evaluation of *RiskScore* results, we were not able to fully elucidate the underlying functional relationships due to the lack of other markers to clearly characterize functional and/or exhaustion states. Hence comprehensive profiling of cell functional states demands the ongoing effort to expand and validate the panel of antibodies used for staining. This study represents a multi-scale characterization of spatial heterogeneity in molecular states, yet their crosstalk at genetic level is still not fully understood. Advanced omics technology such as single-cell RNA sequencing enables a deeper insight into genetic heterogeneity, therefore facilitate a comprehensive mapping of cellular biology when integrated with molecular profiling.662 Finally, there is a considerable interest in the use of image analysis to distill subtle features from pathology samples to predict clinical endpoints (37–41). In this study, tissue specimens were collected from surgical resections after the therapy. This prevented us from searching predictive signals.

## Conclusion

We employed an unbiased, quantitative spatial analysis to determine how tumor and immune components interact in responding and nonresponding HCC tumors. The proposed framework represents a novel application of multiplexed imaging in translational medicine and has potential for initialization and validation of computational immuno-oncology models (42–45).

## Data Availability Statement

The datasets generated or analyzed during this work are available from the corresponding author on reasonable request. The codes for computational methods are made available at https://github.com/popellab/HCC-IMC-processing-pipeline as well as an executable capsule on Code Ocean at https://codeocean.com/capsule/7308874/tree.

## Ethics Statement

The studies involving human participants were reviewed and approved by Institutional review board of Johns Hopkins University. The ethics committee waived the requirement of written informed consent for participation.

## Author Contributions

HM and WH designed the workflow. WH implemented the image processing pipeline. HM implemented the spatial analysis, performed the statistical tests, produced the figures, and drafted the manuscript. WH conducted imaging mass cytometry. All authors contributed to the data interpretations and critically edited the manuscript. All authors contributed to the article and approved the submitted version.

## Funding

Bristol-Myers Squibb (to MY). Exelixis (to MY and WH). National Cancer Institute Specialized Program of Research Excellence (SPORE) in Gastrointestinal Cancers grant P50 CA062924 (to MY and WH). Passano Foundation (to MY). National Institutes of Health grants R01CA138264 and U01CA212007 (to AP). Emerson Collective Cancer Research Fund grant 640183 (to WH). The funders were not involved in the study design, collection, analysis, interpretation of data, the writing of this article or the decision to submit it for publication.

## Conflict of Interest

WH is a co-inventor of patents with potential for receiving royalties from Rodeo Therapeutics. WH is a consultant for Exelixis and receives research funding from Sanofi. MY reports receiving research grants from Incyte, Bristol-Myers Squibb, and Exelixis, and is a consultant for AstraZeneca, Eisai, Exelixis, and Genentech. AP is a consultant to AsclepiX Therapeutics and CytomX Therapeutics. He is the founder and Chief Scientific Advisor of AsclepiX Therapeutics. AP receives research grants from AstraZeneca and Boehringer Ingelheim. The terms of these arrangements are being managed by the Johns Hopkins University in accordance with its conflict-of-interest policies.

The remaining author declares that the research was conducted in the absence of any commercial or financial relationships that could be construed as a potential conflict of interest.

## Publisher’s Note

All claims expressed in this article are solely those of the authors and do not necessarily represent those of their affiliated organizations, or those of the publisher, the editors and the reviewers. Any product that may be evaluated in this article, or claim that may be made by its manufacturer, is not guaranteed or endorsed by the publisher.
