# Supplementary material for: Multi-Scale Spatial Analysis of the Tumor Microenvironment Reveals Features of Cabozantinib and Nivolumab Efficacy in Hepatocellular Carcinoma
Source: Front Immunol. 2022 May 13;13:892250. doi: 10.3389/fimmu.2022.892250 (PMC9136005; doi:10.3389/fimmu.2022.892250)
Supplement: Supplementary file 1 [file DataSheet_1.pdf]

Frontiers in Immunology

Supplementary Materials for

Multi-scale Spatial Analysis of the Tumor Microenvironment Reveals Features of Cabozantinib and Nivolumab Efficacy in Hepatocellular Carcinoma

Haoyang Mi, Won Jin Ho, Mark Yarchoan, Aleksander S. Popel

*Corresponding authors. Email: hmi1@jhmi.edu (H.M.), mark.yarchoan@jhmi.edu (M.Y.)

The PDF files includes:

Technical details of image processing pipelines, spatial analysis, and statistical analysis.

Fig. S1. t-SNE maps for each of the 27-marker panel used for cell lineaging. Cells were visualized as dots and color coded by the normalized expression level of the corresponding marker.

Fig. S2. Voronoi tessellation maps for each tumor region core grouped to responders and non-responders. Polygons were color coded based on associated unique cell types.

Fig. S3. Voronoi tessellation maps for each tumor region core grouped to responders and non-responders. Polygons were color coded based on associated general cell types.

Fig. S4. Protein-protein spatial interaction heatmap describing the z-score between each given pair of proteins in all patients.

Fig. S5. Point patterns describing the relative distance to tumor-immune boundaries and infiltration profiles for all compartmentalized cores (except core 8 and core 22).

Fig. S6. Detailing the compartment analysis.

Fig. S7. Detailing the mixing analysis.

Fig. S8. Detailing the network analysis.

Table. S1. Baseline characteristics of the study participants.

Table. S2. Number of individuals with treatment-related AEs during the neoadjuvant treatment period.

Table. S3. Density statistics for all cell types.

Table. S4. Correlations between different cell types with HCC/hepatocytes.

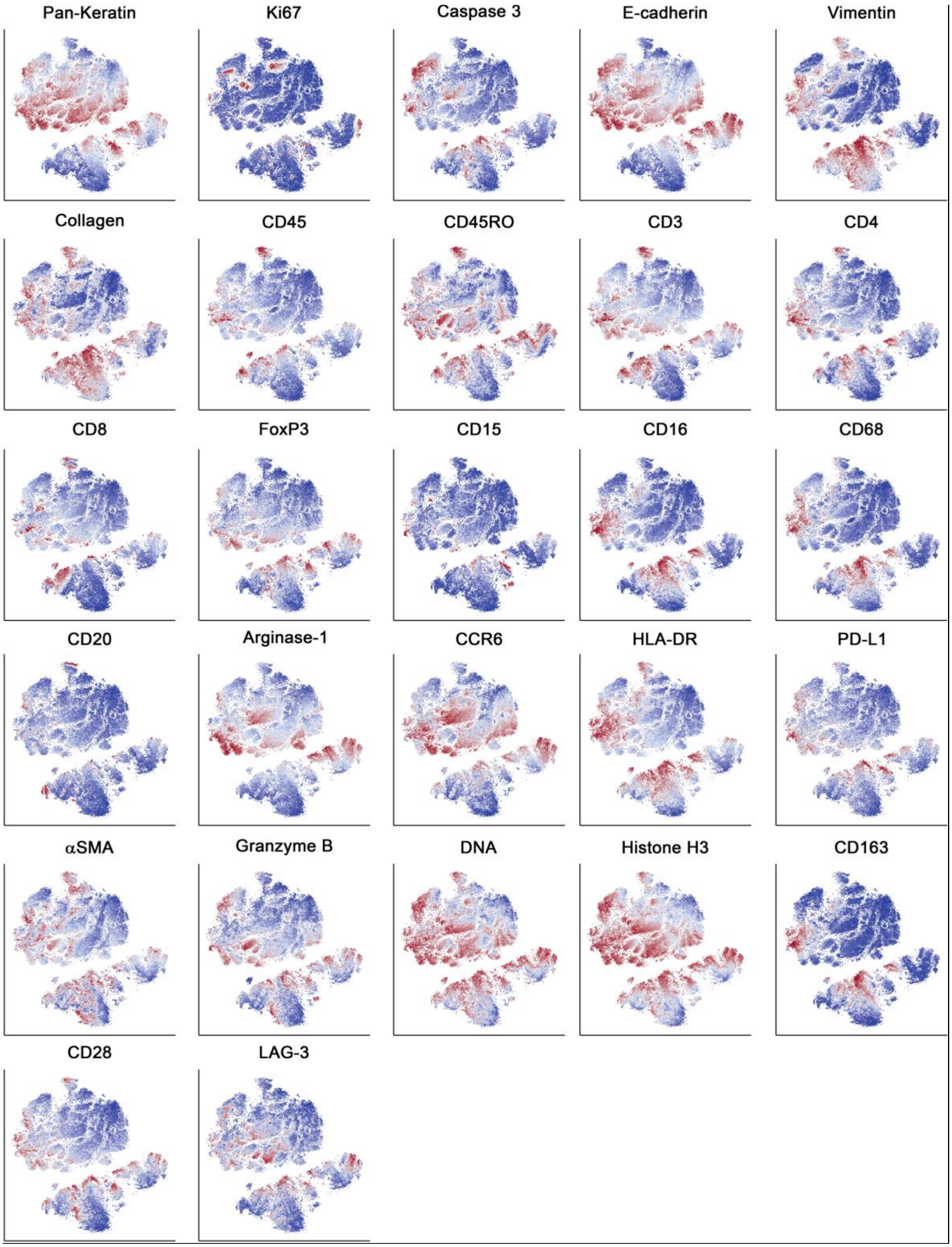

Fig. S1. t-SNE maps for each of the 27-marker panel used for cell lineaging. Cells were visualized as dots and color coded by the normalized expression level of the corresponding marker.

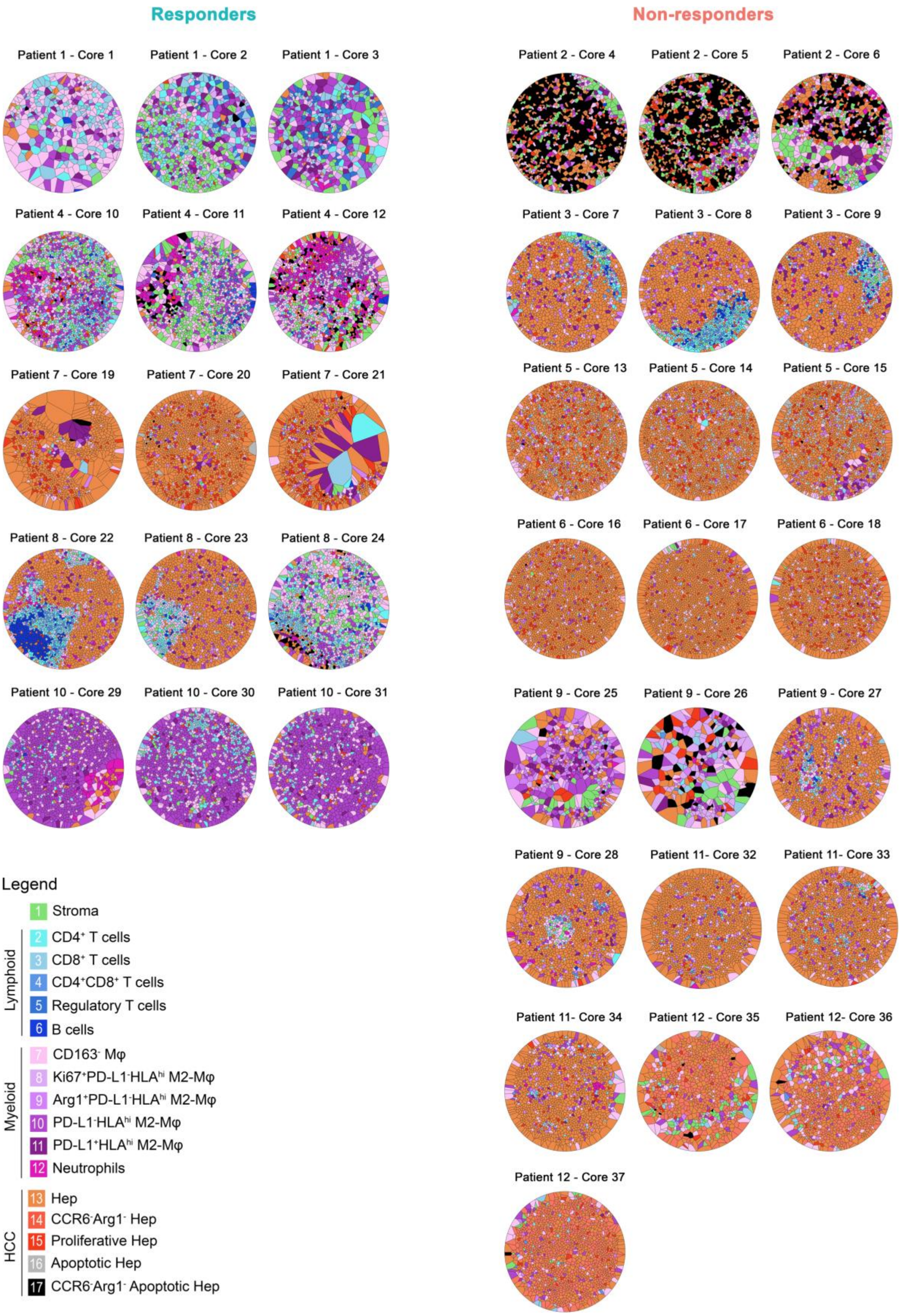

Responders
Non-responders
Patient 1 - Core 1
Patient 1 - Core 2
Patient 1 - Core 3
Patient 2 - Core 4
Patient 2 - Core 5
Patient 2 - Core 6
Patient 4 - Core 10
Patient 4 - Core 11
Patient 4 - Core 12
Patient 3 - Core 7
Patient 3 - Core 8
Patient 3 - Core 9
Patient 7 - Core 19
Patient 7 - Core 20
Patient 7 - Core 21
Patient 5 - Core 13
Patient 5 - Core 14
Patient 5 - Core 15
Patient 8 - Core 22
Patient 8 - Core 23
Patient 8 - Core 24
Patient 6 - Core 16
Patient 6 - Core 17
Patient 6 - Core 18
Patient 10 - Core 29
Patient 10 - Core 30
Patient 10 - Core 31
Patient 9 - Core 25
Patient 9 - Core 26
Patient 9 - Core 27
Patient 9 - Core 28
Patient 11- Core 32
Patient 11- Core 33
Patient 11- Core 34
Patient 12 - Core 35
Patient 12- Core 36
Patient 12 - Core 37
Legend
1 Stroma
Lymphoid
2 CD4$^+$ T cells
3 CD8$^+$ T cells
4 CD4$^+$CD8$^+$ T cells
5 Regulatory T cells
6 B cells
Myeloid
7 CD163$^-$ Mφ
8 Ki67$^+$PD-L1$^-$HLA^{hi} M2-Mφ
9 Arg1$^+$PD-L1$^-$HLA^{hi} M2-Mφ
10 PD-L1$^-$HLA^{hi} M2-Mφ
11 PD-L1$^+$HLA^{hi} M2-Mφ
12 Neutrophils
HCC
13 Hep
14 CCR6$^-$Arg1$^-$ Hep
15 Proliferative Hep
16 Apoptotic Hep
17 CCR6$^-$Arg1$^-$ Apoptotic Hep

Fig. S2. Voronoi tessellation maps for each tumor region core grouped to responders and non-responders. Polygons were color coded based on associated unique cell types.

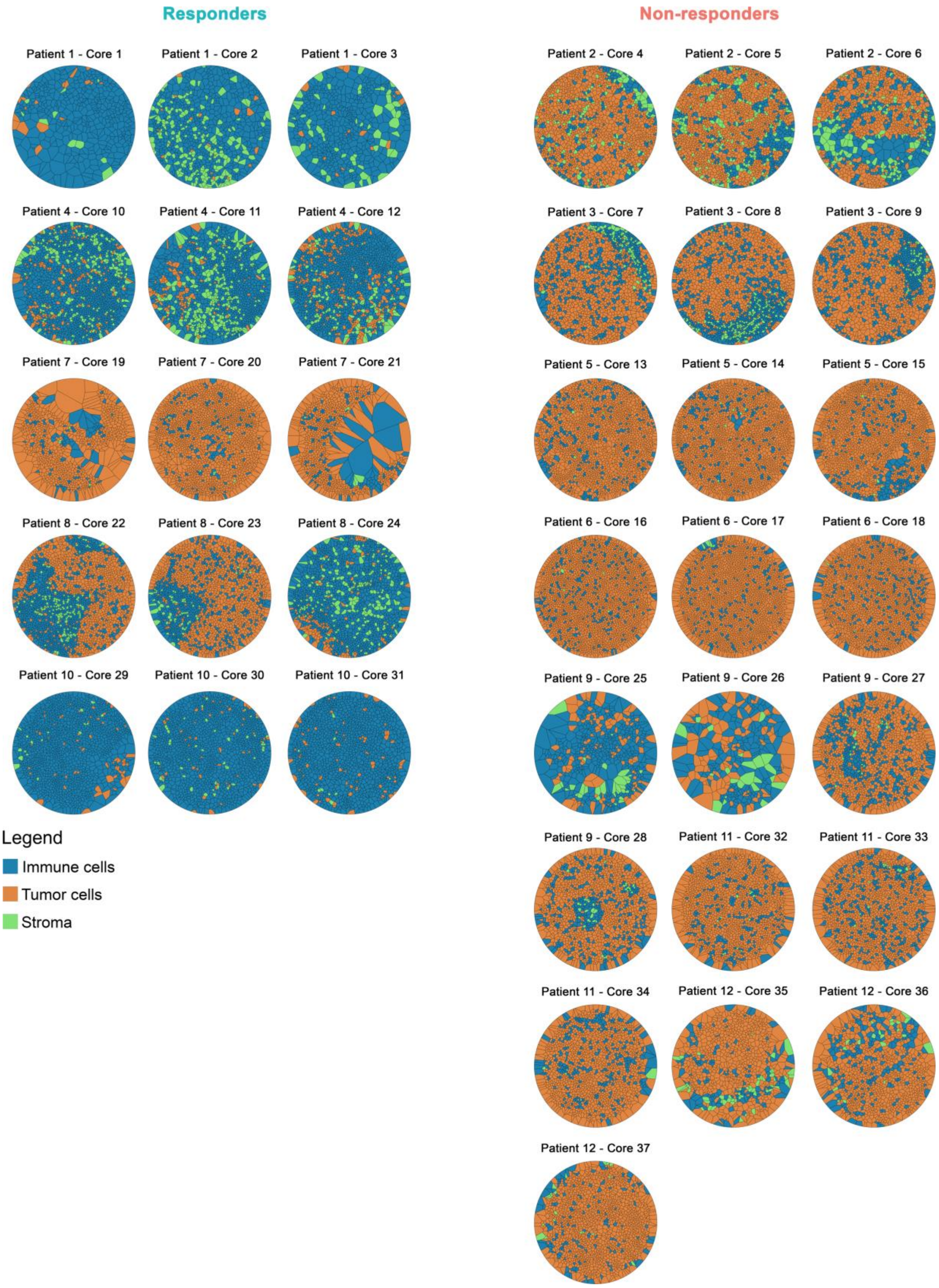
Responders
Non-responders
Patient 1 - Core 1
Patient 1 - Core 2
Patient 1 - Core 3
Patient 4 - Core 10
Patient 4 - Core 11
Patient 4 - Core 12
Patient 7 - Core 19
Patient 7 - Core 20
Patient 7 - Core 21
Patient 8 - Core 22
Patient 8 - Core 23
Patient 8 - Core 24
Patient 10 - Core 29
Patient 10 - Core 30
Patient 10 - Core 31
Patient 2 - Core 4
Patient 2 - Core 5
Patient 2 - Core 6
Patient 3 - Core 7
Patient 3 - Core 8
Patient 3 - Core 9
Patient 5 - Core 13
Patient 5 - Core 14
Patient 5 - Core 15
Patient 6 - Core 16
Patient 6 - Core 17
Patient 6 - Core 18
Patient 9 - Core 25
Patient 9 - Core 26
Patient 9 - Core 27
Patient 9 - Core 28
Patient 11 - Core 32
Patient 11 - Core 33
Patient 11 - Core 34
Patient 12 - Core 35
Patient 12 - Core 36
Patient 12 - Core 37
Legend
Immune cells
Tumor cells
Stroma

Fig. S3. Voronoi tessellation maps for each tumor region core grouped to responders and non-responders. Polygons were color coded based on associated general cell types.

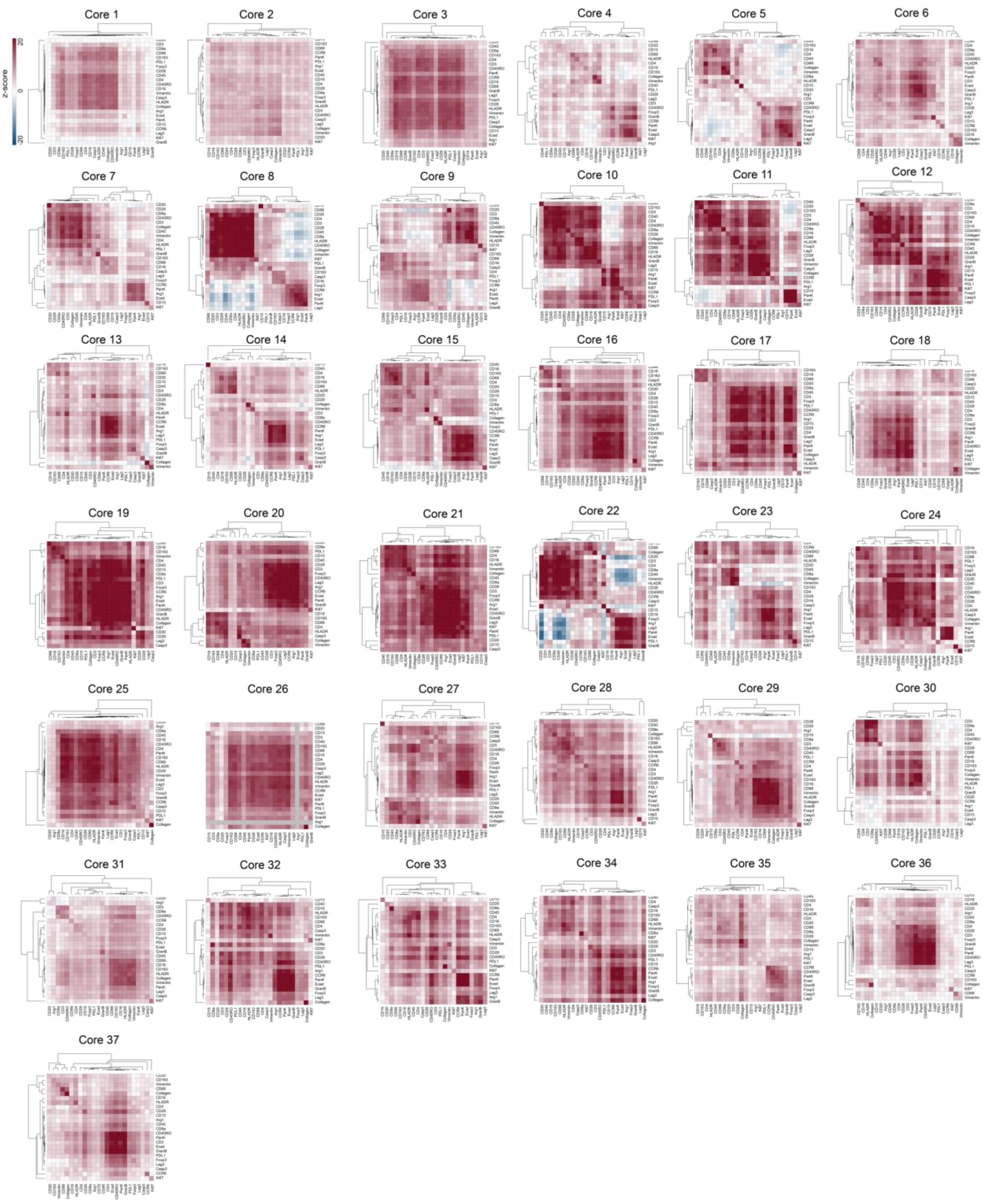

Fig. S4. Protein-protein spatial interaction heatmap describing the z-score between each given pair of protein in all patients.

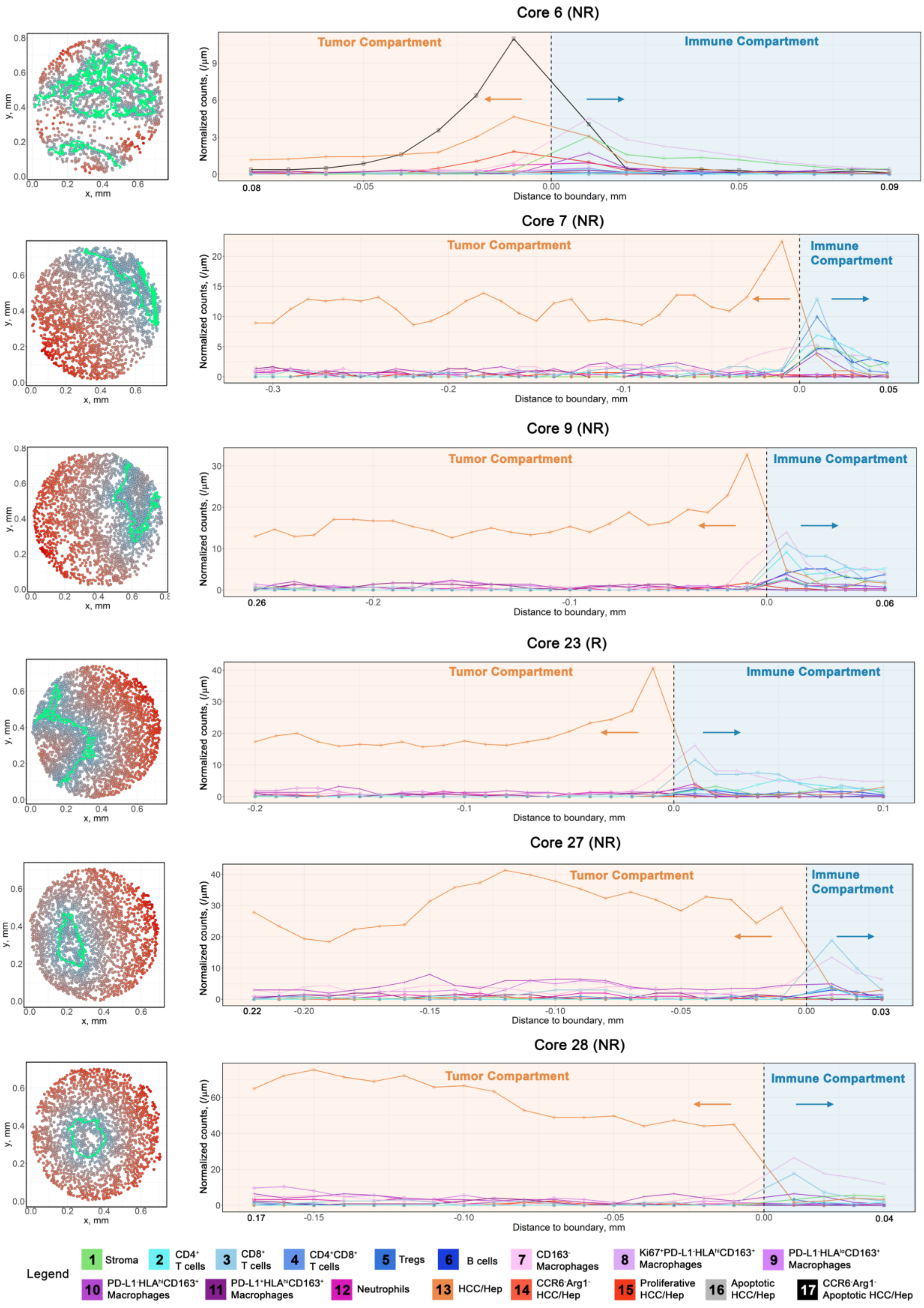

Core 6 (NR)
Tumor Compartment
Immune Compartment
Normalized counts, (/μm)
Distance to boundary, mm
Core 7 (NR)
Core 9 (NR)
Core 23 (R)
Core 27 (NR)
Core 28 (NR)
x, mm
y, mm
Legend
1 Stroma
2 CD4+ T cells
3 CD8+ T cells
4 CD4+CD8+ T cells
5 Tregs
6 B cells
7 CD163- Macrophages
8 Ki67+PD-L1-HLAhiCD163+ Macrophages
9 PD-L1-HLAloCD163+ Macrophages
10 PD-L1-HLAhiCD163+ Macrophages
11 PD-L1+HLAhiCD163+ Macrophages
12 Neutrophils
13 HCC/Hep
14 CCR6-Arg1- HCC/Hep
15 Proliferative HCC/Hep
16 Apoptotic HCC/Hep
17 CCR6-Arg1- Apoptotic HCC/Hep

Fig. S5. Point patterns describing the relative distance to tumor-immune boundaries and infiltration profiles for all compartmentalized cores (except core 8 and core 22).

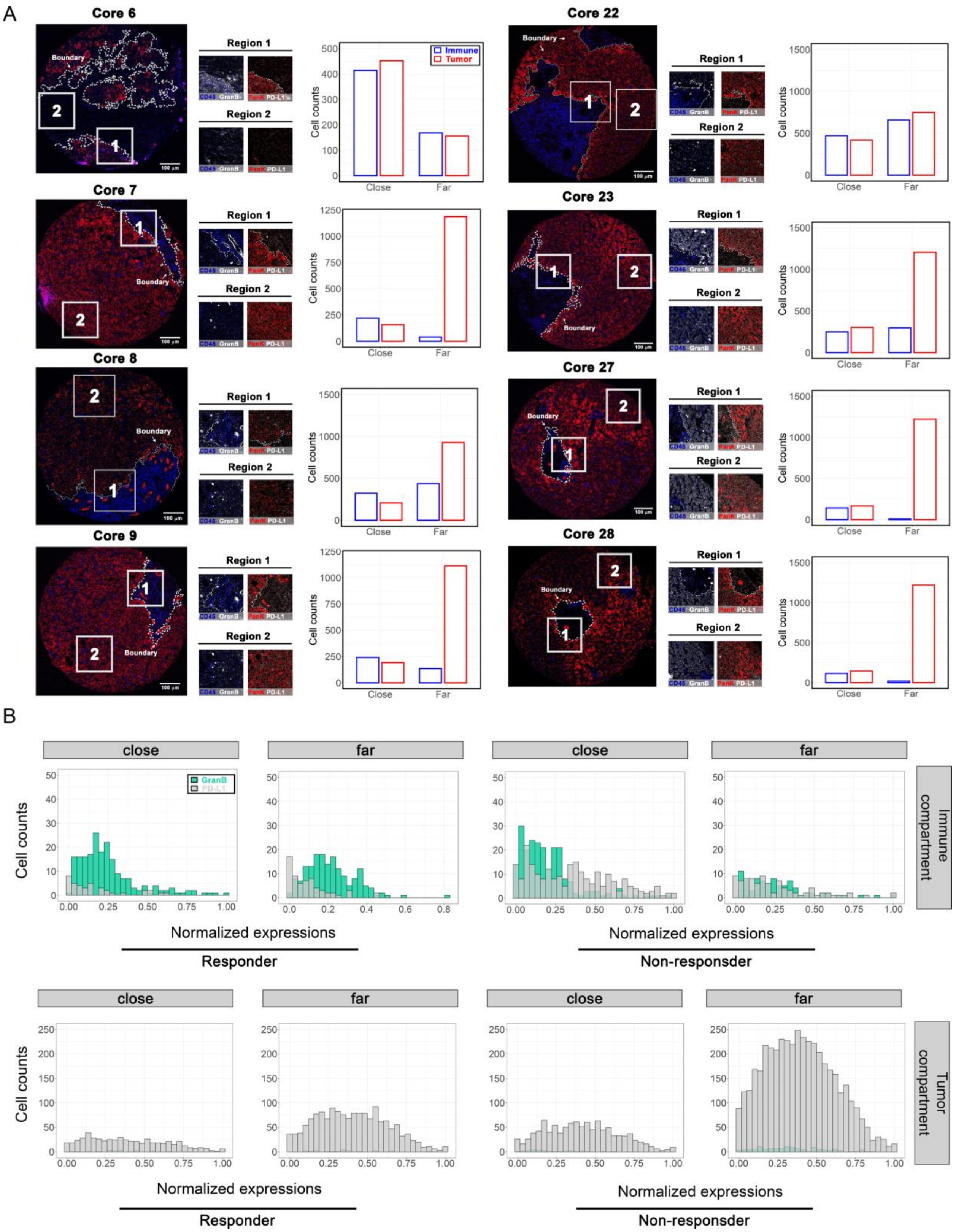

Fig. S6. Detailing the compartment analysis. For each core, two regions exemplifying the staining contexture near and distant to the tumor-immune boundary were selected. For both regions, lineage and functional markers were

simultaneously overlaid. Tumor and immune cell counts at regions close to and far from the boundary were also computed. **(B)** Histograms of LAG-3 and PD-L1 expressions adjacent and distant to the tumor-immune boundary at tumor and immune compartment, grouped by responders and non-responders.

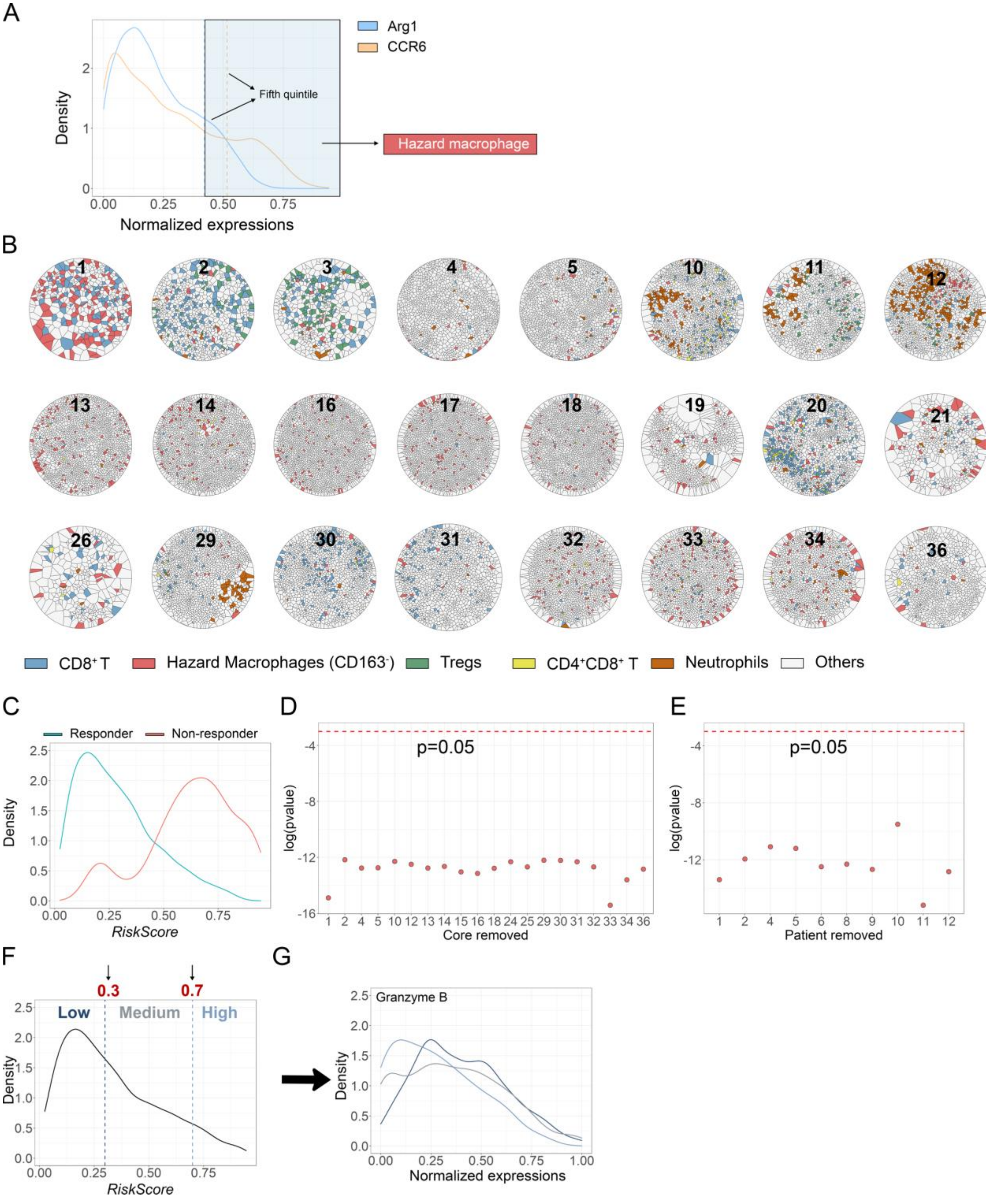

Fig. S7. Detailing the mixing analysis. (A) Density plot of normalized Arg1 and CCR6 expressions on CD163$^-$ macrophages. Dashed lines indicated cut-off thresholds to define up-regulated group. **(B)** Voronoi tessellations depicting spatial proximity between hazard macrophages to CCR6$^-$ expressing cell types for all *mixed* cores. **(C)** Density of *RiskScore* reflected two distinct populations associated with responders and non-responders. **(D)** Dot plot of p-values for the *RiskScore* between responders and non-responders across 20 iterations of individual core

exclusion. Differences between patient groups were modeled with a linear mixed-effects model taking a patient identifier as a random effect and p values were derived using Satterthwaite's degrees of freedom method**. (F)** CD8^{+} T cells were further discriminated to three groups: low risk (*RiskScore* < 0.3), medium risk (0.3 ≤ *RiskScore* < 0.7), and high risk (0.7 ≤ *RiskScore*). **(G)** Density of Granzyme B expressions for each risk group.

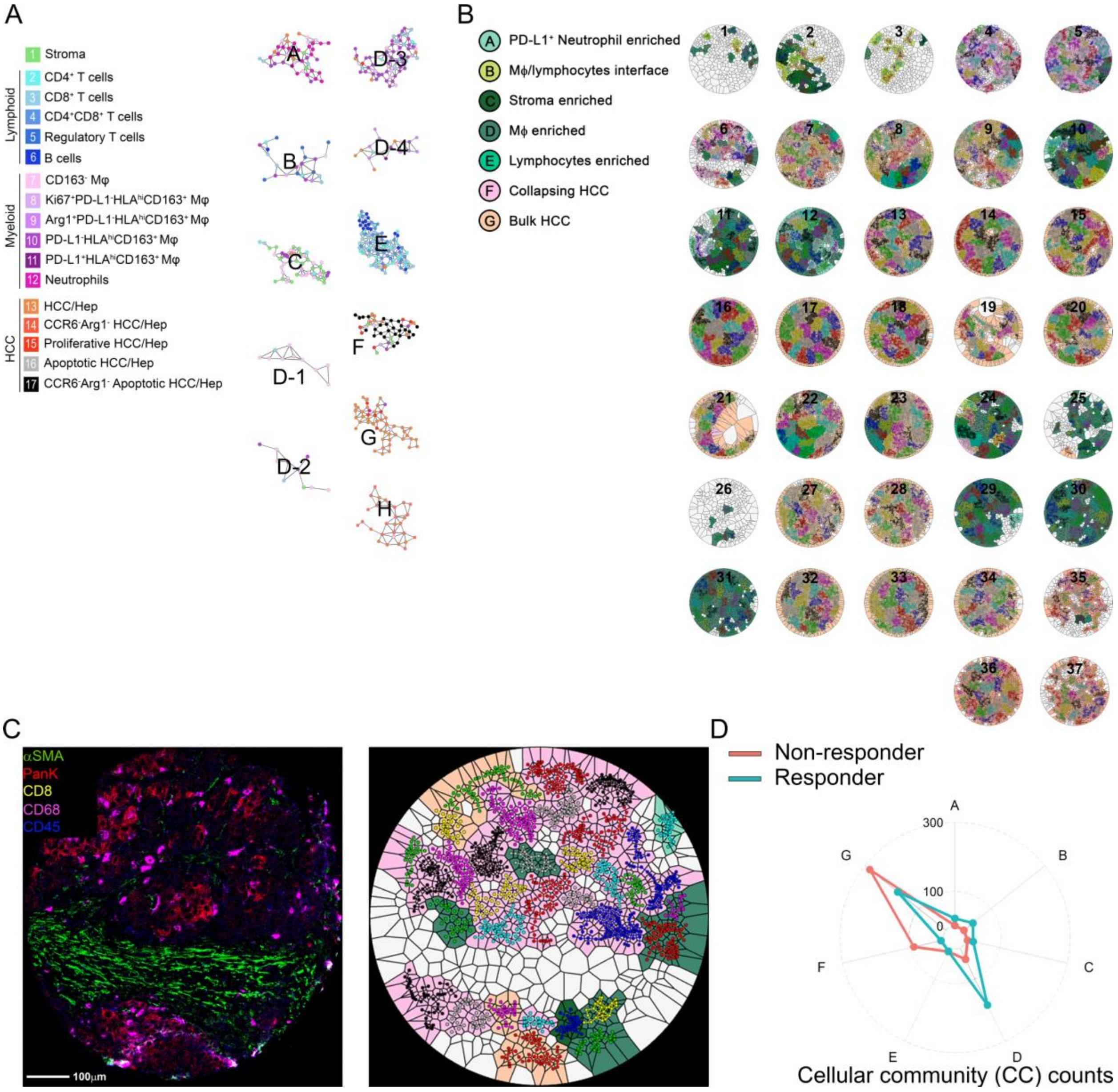

Fig. S8. Detailing the characterization of hazard macrophages and *RiskScore*. (A) Exemplar cellular communities (CCs) depicting each unique CC type, where nodes represent cells and links reflect spatial proximity (Euclidean distance ≤ 20 μm). **(B)** Distribution of CCs in all tumor region core. Polygon colors indicate the associated CC type; node and edge colors were set to highlight the structure of each CC from others. **(C)** Multi-channel rendered multiplex image and Voronoi tessellations for visual validation on network analysis. **(D)** Radar

plot summarizes each CC type quantity in responders versus non-responders. Legend for (B) is provided to interpret the color codes for polygons in (B) and (C). Nodes are colored solely for distinction and without biological meaning.

**Charcteristics**	**N = 15**
**Age in years, median (range)**	64 (41 - 78)
**Sex, N (%)**	
Male	10 (67%)
Female	5 (33%)
Race/Ethnicity N (%)	
White, non-Hispanic	8 (53%)
Hispanic	1 (7%)
Black	5 (33%)
Asian	1 (7)
**Etiology, N (%)**	
Hepatitis B	3 (20%)
Hepatitis C	4 (27%)
Non-Viral	8 (53%)
NASH-/NAFLD	4 (27%)
Alcohol	1 (7%)
Other or Unknown	3 (20%)
**AFP (ng/mL), N (%)**	
<400	20 (44%)
≥400	25 (54%)
≥20000	1 (2%)
**Child-Pugh Score, N (%)**	
A5	13 (87%)
A6	2 (13%)
**MELD Score, median (range)**	7 (6- 10)
**Diameter of large lesion (cm), N (%)**	
<5	1 (7%)
5- 10	8 (53%)
>10	6 (40%)
**Tumor features, N (%)**	
Multinodular	6 (40%)
Portal vein invasion	4 (27%)
Infiltrate	9 (60%)
Adenopathy	1 (7)

Table. S1. Baseline characteristics of the study participants.

Toxicity	Grade 1/2	Grade 3/4	Total (N = 15)
**Any**	14	2	14
**Cardio-renal**			
Fatigue	2	0	2 (13.3%)
Creatine, elevated	1	0	1 (6.7%)
**Constitutional**			
Fatigue	8 (53%)	0	8 (53.3%)
Weight loss	1 (7%)	0	1 (6.7%)
**Dermatologic**			
Palmar-plantar erythrodysesthesia syndrome	2	0	2 (13.3%)
Rash	2	0	2 (13.3%)
Skin hypopigmentation	1	0	1 (6.7%)
**Ears/Nose/Throat**			
Hoarseness	2	0	2 (13.3%)
**Gastrointestinal**			
Abdominal pain	1	0	1 (6.7%)
Anorexia	2	0	2 (13.3%)
Bloating	1	0	1 6.7%)
Diarrhea	2	0	2 (13.3%)
Dry mouth	1	0	1 6.7%)
Dysgeusia	1	0	1 6.7%)
Nausea	5	0	5 (33.3%)
Oral dysesthesia	1	0	1 6.7%)
Mucositis	1	0	1 6.7%)
Vomiting	3	0	3 (20.0%)
ALT, elevated	1	0	1 6.7%)
Autoimmune Hepatitis	0	1	1 (6.7%)
**Musculoskeletal**	7 (6- 10)		
Myositis			
**Neurological and psychiatric**			
Insomnia	1	0	1 (6.7%)
Myasthenia gravis	0	1	1 (6.7%)

Table. S2. Number of individuals with treatment related adverse events (AEs) during the neoadjuvant treatment preriod. AEs are listed as the most severe treatment-related AE reached for each individual patient from the start of the therapy up to 100 after the last dose of the treatment.

Cell type	Observed density in specimen (cells/mm^2)		
	Overall (mean ± SD, range)	Responders (mean ± SD, range)	Non-responders (mean ± SD, range)
**Stroma**	104.04 ± 147.99 (1.99 – 586.88)	160.22 ± 189.02 (3.98 – 586.88)	65.74 ± 99.66 (1.99 – 379.98)
**LYMPHOID**			
**CD4$^+$ T cell**	78.90 ± 117.13 (1.99 – 511.29)	127.32 ± 111.61 (1.99 – 376.00)	46.61 ± 111.76 (1.99 – 511.29)
**CD8$^+$ T cell**	128,94 ± 153.95 (9.95 – 668.45)	222.68 ± 195.90 (9.95 – 668.45)	65.02 ± 66.96 (9.95 – 232.76)
**CD4$^+$CD8$^+$ T cell**	35.88 ± 61.39 (1.99 – 236.74)	48.41 ± 73.63 (1.99 – 230.77)	29.95 ± 55.93 (1.99 – 236.74)
**Tregs**	67.14 ± 64.80 (1.99 – 173.08)	76.45 ± 63.95 (9.95 – 173.08)	1.99 ± NA (1.99 – 1.99)
**B cell**	58.9 ± 131.0 (1.99 – 602.80)	78.67 ± 175.96 (1.99 – 602.80)	37.20 ± 52.19 (1.99 – 157.17)
**MYELOID**			
**CD163$^-$ Mϕ**	355.57 ± 262.21 (49.74 – 1197.64)	505.45 ± 352.52 (99.47 – 1197.64)	153.55 ± 60.87 (49.74 – 449.61)
**Ki67$^+$PDL1$^-$HLA^{hi} M2- Mϕ**	23.81 ± 38.84 (1.99 – 192.98)	30.17 ± 29.36 (3.98 – 95.49)	19.56 ± 44.37 (1.99 – 192.98)
**Arg1$^+$PDL1$^-$HLA^{hi} M2- Mϕ**	80.24 ± 68.20 (5.97 – 306.37)	44.48 ± 46.04 (5.97 – 151.20)	103.00 ± 71.03 (11.94 – 306.37)
**PDL1$^-$HLA^{hi} M2- Mϕ**	361.00 ± 529.70 (45.76 – 2240.11)	665.27 ± 741.02 (45.76 – 2240.11)	153.55 ± 60.87 (71.62 – 268.57)
**PDL1$^+$HLA^{hi} M2- Mϕ**	40.23 ± 33.98 (1.99 – 131.30)	49.74 ± 27.99 (11.94 – 107.43)	33.44 ± 36.82 (1.99 – 131.30)
**Neutrophil**	58.47 ± 115.90 (3.98 – 664.47)	106.86 ± 175.48 (3.98 – 664.47)	27.67 ± 27.78 (3.99 – 83.56)
**HCC**			
**Generic HCC/hepatocytes**	1489.55 ± 1322.71 (25.86 – 4261.37)	743.25 ± 997.10 (25.86 – 2994.10)	1998.39 ± 1291.27 (95.49 – 4261.37)

**CCR6⁻Arg1⁻ HCC/hepatocytes**	139.41 ± 376.24 (1.99 – 1710.92)	19.65 ± 17.06 (1.99 – 47.75)	192.64 ± 445.31 (1.99 – 1710.92)
**HCC/hepatocytes Proliferative**	125.39 ± 139.84 (3.98 – 431.71)	96.20 ± 140.44 (3.98 – 431.71)	143.96 ± 139.47 (15.92 – 395.90)
**Apoptotic HCC/hepatocytes**	88.96 ± 163.63 (1.99 – 499.35)	17.90 ± 19.69 (3.98 – 31.83)	100.80 ± 174.75 (1.99 – 499.35)
**CCR6⁻Arg1⁻ Apoptotic HCC/hepatocytes**	206.00 ± 478.45 (1.99 – 1617.41)	51.53 ± 78.81 (1.99 – 240.72)	334.72 ± 626.23 (1.99 – 1617.41)

Table. S3. Density statistics for all cell types.

Core	Response	$CD4^+$ T	$CD8^+$ T	Treg	B cell	$CD163^-$ macrophage	$CD163^+$ macrophage
6	NR	0.1714	0.2353	0.9538	0.2583	0.8875	**$9.8358e^{-6}$**
7	NR	0.4364	0.4804	0.1611	0.2583	0.2583	0.3233
8	NR	**0.0191**	**0.0071**	0.5083	**0.0013**	**0.0256**	0.2583
9	NR	**0.0006**	**0.0426**	0.3518	0.1721	**0.0426**	0.2353
22	R	**$2.9845e^{-7}$**	**$1.0015e^{-8}$**	0.8294	**0.0071**	0.2232	**0.0426**
23	R	0.1763	**0.0318**	0.1454	0.6658	0.1763	0.0714
27	NR	0.6347	0.0834	0.9721	0.6852	0.8311	0.0834
28	NR	0.1763	0.4265	**0.0318**	0.0834	0.0834	**0.0098**

Table. S4. Correlations between different cell types with HCC/hepatocytes. Color code: black (not significant); red (significant with positive correlation); blue (significant with negative correlation). Here, HCC/hepatocytes evaluated represents a collection of all HCC/hepatocytes (cell type 13, 14, 15, 16, 17). Double positive T cells ($CD4^+CD8^+$) was absent from evaluable cores; neutrophils only reside in core 6, therefore they were removed from correlation analysis. Macrophage subpopulations were merged to binary phenotypes ($CD163^+$ vs. $CD163^-$) to increase sample sizes. Correlation tests were performed using Wilcoxon rank-sum test with adjustment for multiple comparisons (FDR).
